# Isolation, characterisation and potential applications of a novel bacteriophage targeting beta-lactam-resistant *Staphylococcus saprophyticus*

**DOI:** 10.1038/s41598-026-35899-3

**Published:** 2026-02-05

**Authors:** O. Gopika, Niti Sarat, Maanya Manikandan, S. Sumana, P. C. Parvathi Mohanan, Ajith Madhavan, R. Sandeep Varma, Samiran Mahapatra, Bipin G. Nair, Sanjay Pal

**Affiliations:** 1 Amrita Vishwa Vidyapeetham, Kollam, Kerala 690525 India; 2 Unilever R&D Bangalore, 64, Main Road, Whitefield, Bangalore, 560066 India

**Keywords:** Staphylococcus saprophyticus, Bacteriophages, Opportunistic infections, Antimicrobial resistance, Biofilm, Biotechnology, Microbiology

## Abstract

*Staphylococcus saprophyticus* is a gram-positive commensal characteristically resistant to novobiocin. Clinically, it is associated with urinary tract and other opportunistic infections. But reports on phages against them are scanty, which underlines the importance of the study. We isolated a *Staphylococcus saprophyticus* strain from soiled socks. The strain was characterised through biochemical tests and 16S rDNA ribotyping. Furthermore, the antibiogram revealed that the strain is resistant to penicillinase-resistant beta-lactam antibiotics. A double-stranded DNA temperate phage, ØPh_SS01, was isolated against *S. saprophyticus* from a mixed sewage sample. The phage is stable over a wide range of temperatures, including pH and salt, with 67.08 ± 25 virions/cell following adsorption within 14 min. The potential of ØPh_SS01 was established in Mannitol Salt broth with a 7-log reduction of *S. saprophyticus* over a period of 24 h. Moreover, it inhibited and disrupted biofilm at efficiencies of 77% and 69%, respectively, at an MOI of 100. Studies on adherent immortalised human keratinocytes and urethral cell lines did not result in any significant change in viability. Sequence analysis of ØPh_SS01 depicted a genome size of 47 Kb with 37.36% GC content. A similarity of 91.5% to related entries indicated its novelty. The textile matrices immobilised with phages could reduce the bacterial load by about 7-log, which shows the potential of the phage in infection control.

**Supplementary Information:**

The online version contains supplementary material available at 10.1038/s41598-026-35899-3.

## Introduction

Approximately 500 million people worldwide are affected by urinary tract infections (UTIs), making them one of the most prevalent bacterial infections globally^1,2^. The mortality rate of UTI-infected individuals approximates well over 200 thousand in the time frame between 1990 and 2019^2,3^. *S. saprophyticus* is referred to as the second most frequent causative agent of UTI infections in females^4^. The recurrence of UTI infections caused by *S. saprophyticus* has attracted increasing attention, as it colonises 6.9% of the urogenital tracts of healthy women and leads to secondary recurrent infections, especially uncomplicated UTIs, with a lifetime prevalence of 50–60 as it colonises 6.9% of the urogenital tracts of healthy women and leads to secondary recurrent infections, especially uncomplicated UTIs with a lifetime prevalence of 50–60% in adult women^5,6^. In males, UTIs caused by *S. saprophyticus* are usually confined to elderly people over fifty years of age rather than younger males^7^. The organism causes approximately 5–20% of non-hospital-acquired UTIs, especially in prepubertal females, ruling out the possibility of nosocomial or iatrogenic infections; rather, secondary self-infections are more common^8^. The distribution of *S. saprophyticus* extends from the gastrointestinal tract to the genitourinary tracts, which accounts for the fact that the organism is a commensal that fans out the rectum, urethra, cervix and even urine of healthy women, indicating the plausibility of *S. saprophyticus* acting as a reservoir of self-infection^8^. Beta-lactamase-producing *S. saprophyticus* strains are becoming more prevalent; hence, multidrug-resistant strains are posing a significant threat. All *S. saprophyticus* species are inherently resistant to novobiocin antibiotics because of mutations in the target DNA gyrase, specifically in its B-subunit, disabling the ability of the antibiotic to bind the target and put forth its action^9^. Penicillin resistance is a significant issue among *S. saprophyticus*, extending its resistance even to penicillinase-resistant penicillins such as oxacillin, methicillin, and cloxacillin^10–12^. The biofilm-forming capability of this organism aids its virulence and extended survival, as it has specific adhesion proteins (Aas, UafA, UafB) which enable adherence to uroepithelial cells, supporting enhanced colonisation and biofilm formation^13^. Secretions such as urease, lipase, and elastase enhance survival in urine, causing inflammation and destruction of uroepithelial cells. The action of antibiotics on biofilms is less efficient than that on planktonic cells, yielding the organism’s enhanced survival benefits when they exist as biofilms^14^.

*S. saprophyticus-*mediated uncomplicated UTIs have high recurrence rates with symptoms such as dysuria, urgency and pyuria in females, whereas in males they are characterised by pyelonephritis, urethritis, and prostitis^8,15^. The organism is also responsible for abnormalities in spermatogenesis leading to sterility in males^16^. As a commensal, it is also associated with food spoilage, especially in meat, fish, milk, and milk products. The other major infections associated with *S. saprophyticus* include septicaemia, opportunistic meningitis, implant-associated infections, endocarditis, ocular and skin infections^17,18^. As a native commensal organism of the human body, it is often associated with body malodour^19^. Thus, the UTIs and opportunistic infections caused by *S. saprophyticus* are becoming inconvenient and vexatious; furthermore, the multidrug resistance of this species is also a concern. Bacteriophages (phages) could be employed to mitigate these infections, as they are well-suited to the bill owing to their self-amplification ability and high specificity. With an estimated 10^31^ particles on Earth, bacteriophages are the most plentiful particles on the planet—an unimaginably large number^20^. The most peculiar characteristic of bacteriophages is their extreme specificity to their host. Consequently, phages facilitate the targeted control of bacteria at a level that is below the infectious dosage. The phage replication cycle can be broadly studied under two major cycles viz., the lytic cycle and the lysogenic cycle^21,22^. The lytic cycle involves the entry of the phage genome into the host cell followed by lysis of the host cell, resulting in the release of progeny virions whereas the lysogenic cycle generally involves incorporation of the phage genetic material into the bacterial genome, forming a prophage association^22^. Lytic phages usually follow a lytic cycle in which they undergo obligate lysis of the host cell, whereas temperate phages operate as prophages inside the host genome until the environmental cues trigger a switch to the lytic cycle. The formulation of phage cocktails rather than the use of a single phage against these antibiotic-resistant opportunistic pathogens would be effective in countering them but for cocktail formulation each of the individual phages need to be characterized and studied otherwise it might lead to redundancy or antagonism between them^23–25^. Biological deterrence via phages is sustainable, nontoxic, novel, and translatable. They are safe for humans since they discriminate their target bacteria from mammalian cells (non-targets) owing to their high specificity characterised by the lack of receptors in the former, which is a prerequisite to initiate infection^25,26^. In light of the necessity to implement alternative techniques to mitigate antibiotic-resistant *S. saprophyticus*, the exploration of bacteriophages is warranted due to their efficacy and specificity, as investigated in the described work. Bacteriophages, as highly specialised bacterial viruses, provide a targeted and environmentally sustainable alternative for managing *S. saprophyticus*. Phage application can proficiently lyse bacterial cells, disrupt mature biofilms, and prevent recurrence of infection without disturbing the commensal flora. Employing phages to mitigate *S. saprophyticus* in clinical and environmental settings would be an effective alternative to conventional antibiotic therapy. This strategy represents a sustainable, eco-friendly solution for combating resistant uropathogenic *S. saprophyticus* and thus effectively containing the associated antibiotic resistance. To serve that objective, we employed a coagulase-negative, novobiocin-resistant *S. saprophyticus* strain isolated from the human microbiota to establish the efficacy of ØPh_SS01 isolated from pooled sewage. The phage was characterised for its stability at different temperatures, pH, organic solvents, and saline conditions, and for its ability to reduce planktonic cell formation, besides biofilm formation. Adsorption efficacy, burst size, Restriction Fragment Length Polymorphism (RFLP) profiling, and Whole Genome Sequencing (WGS) analysis were also done to establish the identity, novelty, and lifestyle of the phage. Thus, we propose this project as an initiative and exemplary study for the establishment of bacteriophage-mediated mitigation of opportunistic pathogens and their associated biofilms without the spurious use of antibiotics.

## Results

### Characterisation of the *S. saprophyticus* strain

*Staphylococcus saprophyticus* strain isolated from foetid socks, when cultured in Mannitol Salt Agar (MSA), produced yellow-pigmented, dry, umbonate colonies with serrate margins. The strain isolated was gram-positive, coagulase-negative, urease-positive and novobiocin-resistant. The bacterial isolate was identified via the amplification and sequencing of the 16S rDNA and subsequently compared with sequences in the NCBI GenBank database via BLAST.

### Characterisation of the phage ØPh_SS01

#### Morphological characterisation

**Fig. 1:**
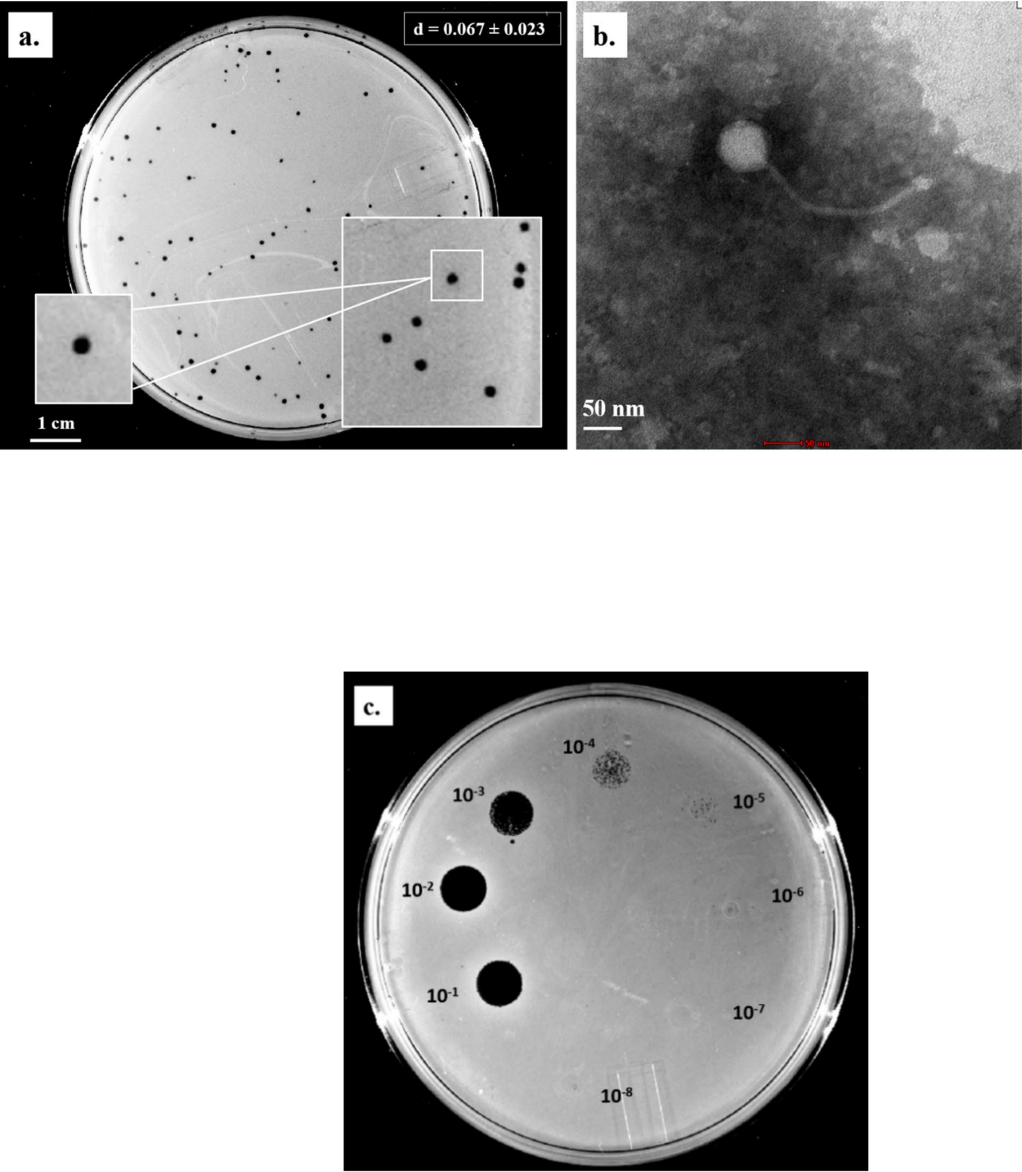
ØPh_SS01 morphological characterisation. Plaque morphology, TEM imaging and spot assay of the phage. (**a**) Plaque morphology of ØPh_SS01 on 1.8% (hard) and 0.35% (soft) agar, (**b**) TEM image of ØPh_SS01, and (**c**) phage spot assay confirming the production of individual plaques, ruling out the absence of bacteriocins.

The plaque morphology and the structure and dimension of the virion were deduced via the agar overlay method and Transmission Electron Microscopy (TEM) imaging, respectively. ØPh_SS01 produced clear, circular, pinpoint, pleomorphic plaques with ragged edges and a plaque diameter of 0.067 ± 0.023 cm, as shown in Fig. 1(a). TEM analysis revealed the complete virion length of 343.2 ± 35.5 nm, the tail length of 269.7 ± 33.8 nm, and the head diameter of 80.18 ± 30.5 nm, as shown in Fig. 1(b) ØPh_SS01, which was isolated against *S. saprophyticus*, demonstrated complete clearance in the spot assay and formed individual plaque-forming units upon dilution, ruling out the involvement of bacteriocins, as depicted in Fig. 1(c).

### Tropism (phage host range)

The host range of the phage ØPh_SS01 was investigated against 12 bacterial strains via the spot assay technique. The clearance (lysis) zones were obtained against *S. ureilyticus*, *S. arlettae*, *S. gallinarum*, *S. epidermidis* and *B. stratosphericus.* The extended infectivity demonstrates the extensive polyvalency and wide-range efficacy of the phage (Table 1).

### Phage stability

**Fig. 2:**
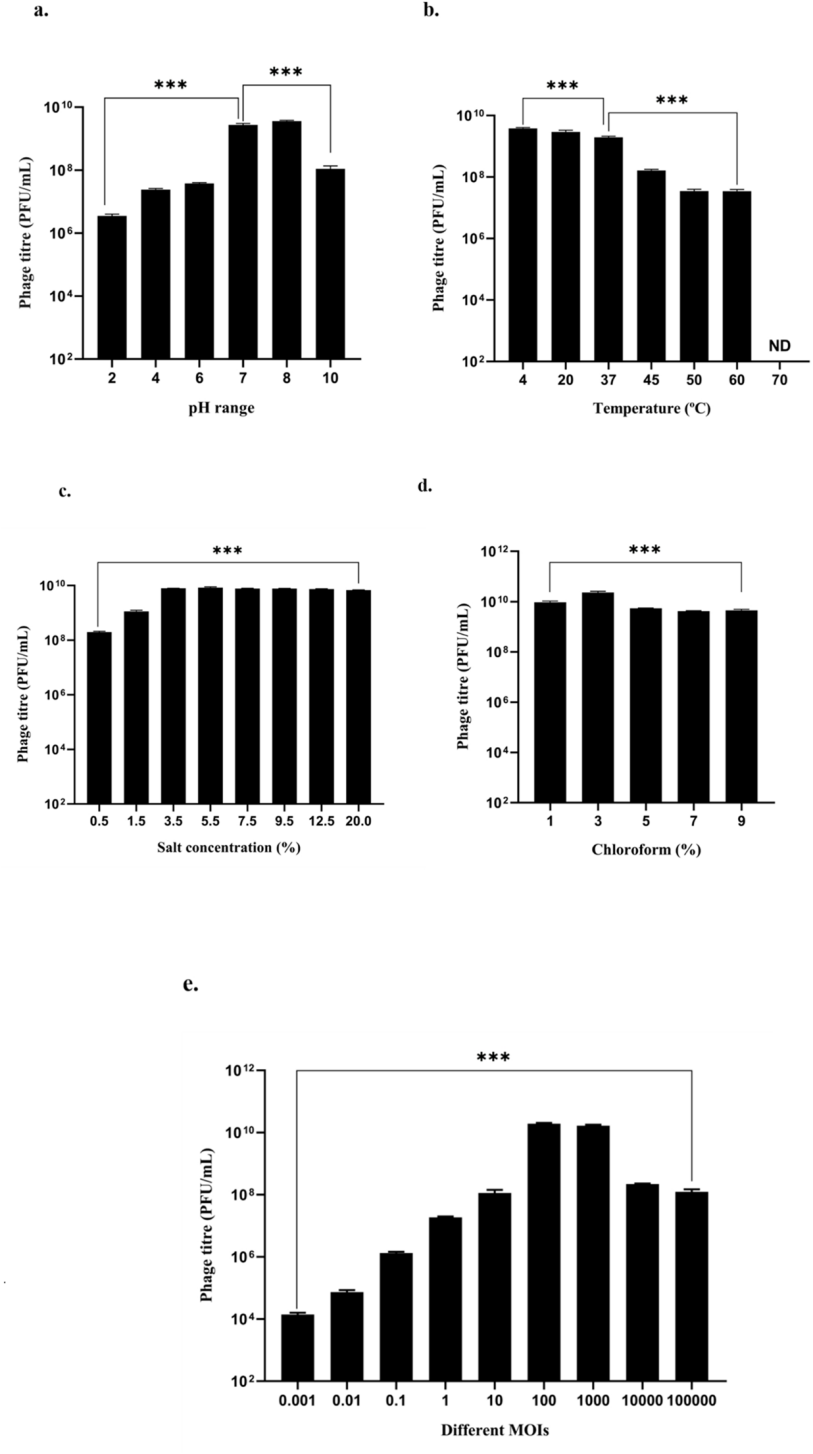
Stability characteristics and optimal MOI of ØPh_SS01. (**a**) pH, (**b**) temperature, (**c**) salt tolerance, (**d**) chloroform tolerance (up to 9%), (**e**) optimal MOI, ***: *p* < 0.001. The error bars represent the standard deviations of the three replicates from independent experiments. ND, not detected.

The effects of different pH, temperature, chloroform, and salt concentrations on ØPh_SS01 were investigated. ØPh_SS01 showed maximum stability at pH 7 and minimum stability at pH 2, whereas at pH 4, 6 and 10, it presented moderate stability, as shown in Fig. 2(a). A temperature stability study indicated considerable stability of ØPh_SS01 over a temperature range of 4–37 °C. Exposure to 45 °C led to a 1-log reduction in phage titre, followed by 50 °C and 60 °C, which resulted in an ~ 2-log reduction, and upon exposure to 70 °C, the phage viability was completely retarded, as shown in Fig. 2(b). ØPh_SS01 was highly salt tolerant (up to 20%), as shown in Fig. 2(c). The stability of ØPh_SS01 in organic solvents such as chloroform was determined, and it was extremely stable over concentrations ranging from 1 to 9%, ensuring effective means for phage separation and purification from the host, post-enrichment, rather than the use of expensive techniques such as 0.22 μm syringe filters as indicated in Fig. 2(d). Thus, ØPh_SS01 can withstand moderate acidity, alkalinity, high temperatures, high salinity, and chloroform (organic solvent) treatments.

**Table 1:** Tropism profile of phage ØPh_SS01. The sensitivity and resistance are represented as ‘+’ and ‘-’, respectively.

Bacterial strains	ØPh_SS01 (phage)
*S. saprophyticus* (host)	+
*S. arlettae*	+
*S. gallinarum*	+
*S. ureilyticus*	+
*S. pasteuri*	-
*S. epidermidis*	+
*S. haemolyticus*	-
*S. warneri*	-
*S. aureus* MRSA	-
*B. stratosphericus*	+
*B. safensis*	-
*S. epidermidis* ATCC	-

### Optimal MOI determination

The ratio of phages to bacteria is considered the MOI. The results indicated that ØPh_SS01 generated the maximum number of progenies at a higher MOI of 100 and 1000 with a titre of 20 × 10^9^ PFU/mL. At a lower MOI of 0.001, the titre of the phage was dramatically reduced with a minimum number of progeny virions and a titre of 12 × 10^3^ PFU/mL, as depicted in Fig. 2(e). Moreover, there was a decrease in trend in titre beyond 1000 MOI, viz.; in MOIs 10,000 and 100,000. Thus, ØPh_SS01 works with maximum efficacy at an MOI of 100 and above.

### Adsorption efficacy and burst size (one-step growth curve)

**Fig. 3:**
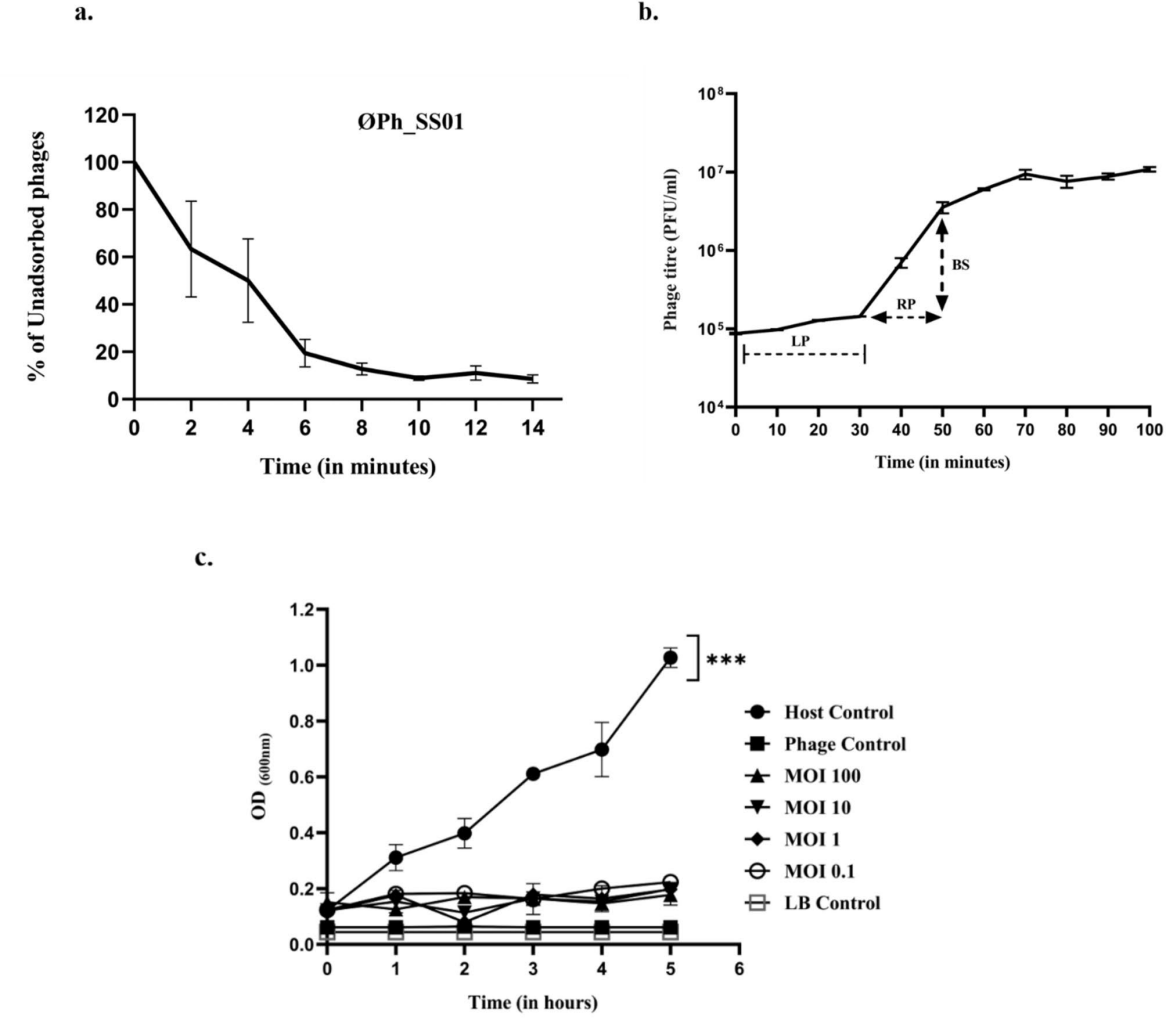
Phage adsorption efficacy, one-step growth curve, and bacteriolytic activity of ØPh_SS01 towards *S. saprophyticus*. (**a**) Adsorption rate, (**b**) one-step growth curve and burst size, the latent period (LP), rise period (RP), and burst size (BS) of ØPh_SS01 are depicted, and (**c**) bacteriolytic efficacy of ØPh_SS01 at different MOI. The bacterial host was significantly reduced by ØPh_SS01 at different MOI, ***: *p* < 0.001. The error bars represent the standard deviation from three replicates from independent experiments.

ØPh_SS01 demonstrated an adsorption efficacy of ~ 91.5% in 14 min. Figure 3(a). The one-step growth curve plot analysis divulged that the latent period of the phage was close to 30 min and the rise period was 20 min with a burst size of 67.08 ± 24.5 virions per cell, as shown in Fig. 3(b).

### Efficiency of plating (EOP)

The efficacies of the bacterial strains that presented tropism with ØPh_SS01 were subjected to EOP analysis (Table 2). All the alternative hosts that cross-reacted with the phage presented lower EOPs (above 1) compared to the original host, which precisely indicated the moderate production efficacy of these bacterial strains in amplifying the phage.

### Molecular characterisation of ØPh_SS01

#### Restriction profile (RFLP), whole genome sequencing (WGS) and annotation of ØPh_SS01

**Fig. 4:**
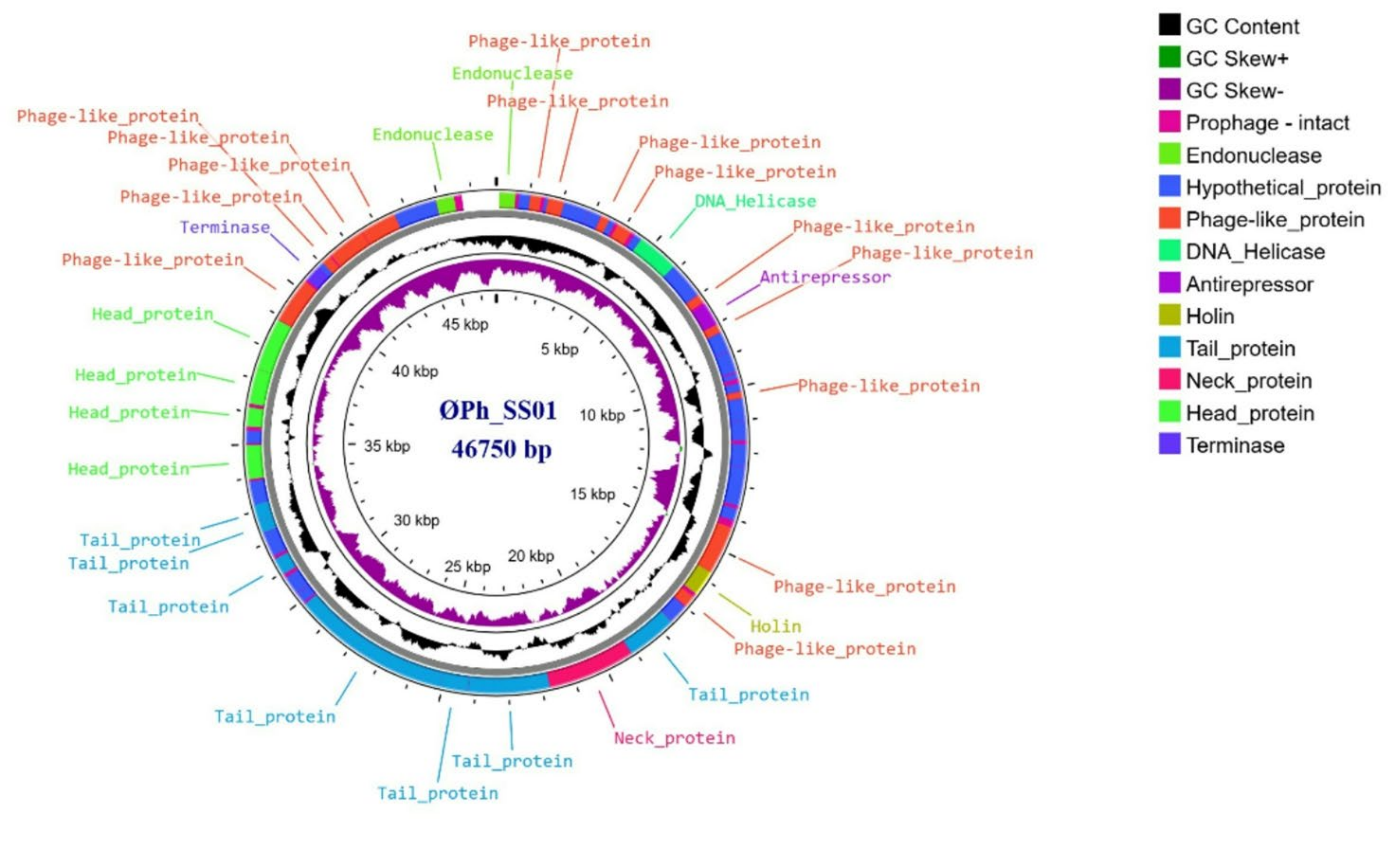
Circular genome map of ØPh_SS01. The map was generated with Proksee genome annotation and visualisation tool. The concentric rings, from the inside to outside, represent GC skew -, GC skew +, and GC content.

The phage ØPh_SS01 was further characterised by restriction digestion with the restriction enzymes (*Eco*RI, *Bam*HI, *Hind*III, *SSp*I, *Kpn*I, *PmI*I, *HpaI)*, which explained that the phage had double-stranded DNA as genetic material with a genome size above 10 Kb. The restriction digestion profile indicated that ØPh_SS01 was sensitive to *Eco*RI, *SSp*I and *Hpa*I but remained resistant to all other restriction enzymes used, as shown in Supplementary Figure S1. The whole genome assembly of ØPh_SS01 resulted in a contig size of 46,750 bp, which showed closest homology to *Staphylococcus* phage TPWB-09 with maximum coverage, an identity of 91.5% and a GC content of 37.36%. The genome hosts 73 ORFs, of which 50 are known and the remaining 23, hypothetical. These unannotated ORFs were further analysed using a homology search approach against the NCBI NT database, which resulted in the annotation of 20 ORFs when compared against a reference *Staphylococcus* phage TPWB-09 strain. The lifestyle of the phage is temperate owing to the presence of anti-repressor, which is considered to be a signature of lysogeny. On phylogenetic analysis, ØPh_SS01 occupied a separate clade when compared to related phages (36 phage sequences), indicating a novel/rare lineage. Moreover, comparative analysis against the entries in the NCBI virus database ØPh_SS01 depicted a maximum similarity of only 91.5% with a *Staphylococcus* phage TPWB-09 (Gen Bank ID: PQ358088.1), confirming it as a new phage species (according to ICTV guidelines and ANI similarity). The genome is organised into functional modules, with genes encoding structural proteins, enzymes, regulatory elements and the remainder into a group with unspecified function, as shown in Fig. 4.

#### Phylogenetic assembly

**Fig. 5:**
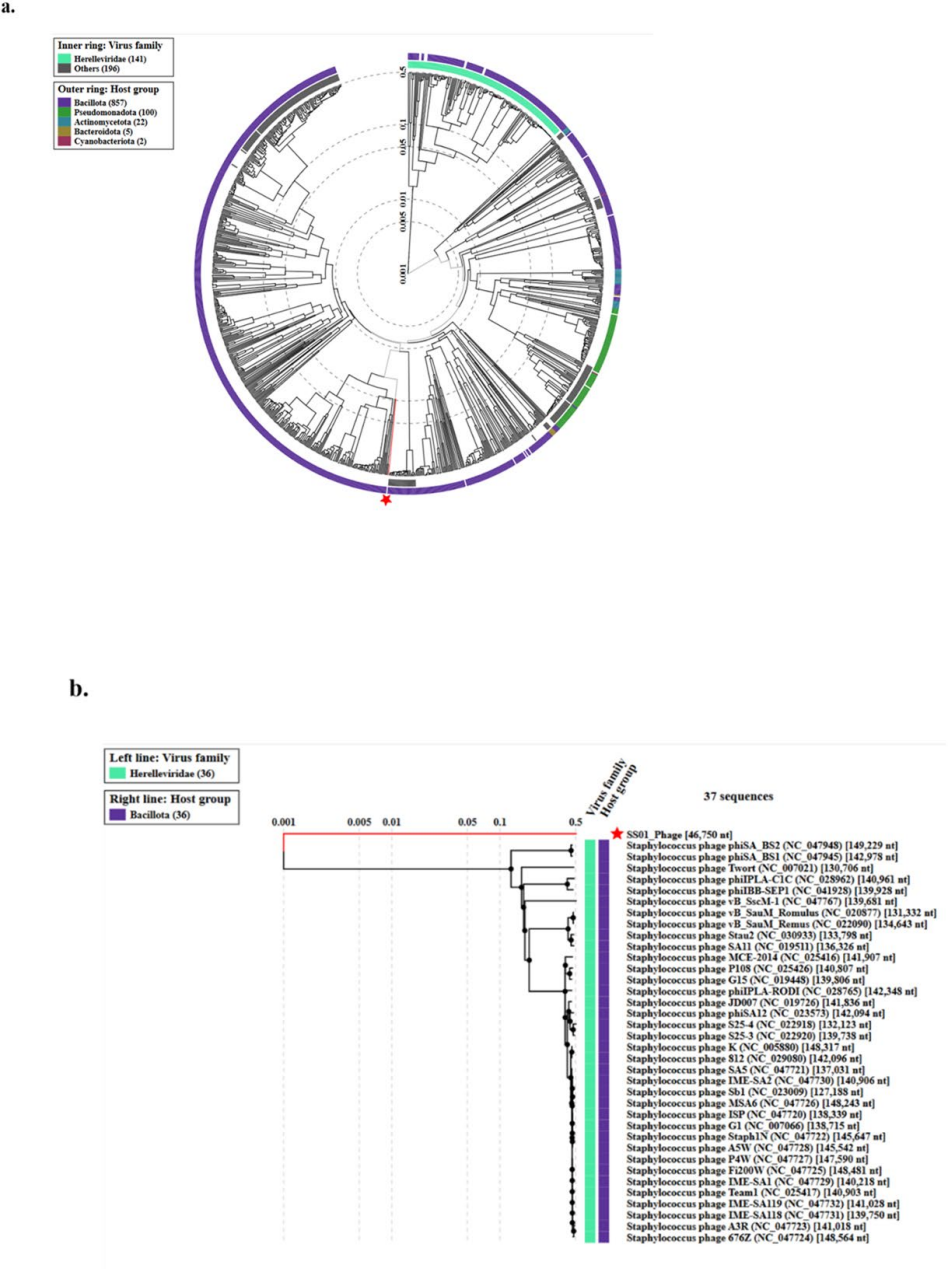
Phylogenetic characterisation of ØPh_SS01. (**a**) A circular dendrogram representing the phylogenetic relationship of ØPh_SS01 with 1008 phage genome sequences. (**b**) A dendrogram constructed with the subset of 36 related phage genome sequences. The green and blue stripes represent the host and phage groups at the family level, respectively.

A proteome-based phylogenetic tree for taxonomic classification was generated using VipTree. On comparing ØPh_SS01 with 1008 identified prokaryotic dsDNA virus genomes, the phage did not show any sequence similarity to any of the virus families included in the database, as shown in Fig. 5a. On comparing with 36 Staphylococcus phages, ØPh_SS01 was placed in a unique clade, depicting evolutionary variation from others, indicating its novelty as represented in Fig. 5b.

### Antibiogram of the bacterial strains

Antibiotic susceptibility profiling of *S. saprophyticus* indicated that the organism was resistant to all beta-lactam antibiotics tested, including penicillinase-resistant penicillins (penicillin G, oxacillin, methicillin, amoxicillin, ampicillin and ticarcillin), third-generation cephalosporins (ceftazidime), fluoroquinolones (ciprofloxacin) and aminocoumarins (novobiocin) but was sensitive to all the other antibiotic classes tested. (Supplementary file, Table S1 and S2).

### Bacteriolytic assay at different MOI

The bactericidal activity of the phage was determined via an in vitro bacteriolytic assay as depicted in Fig. 3(c). The bacteria and phage were mixed at different MOIs, namely 0.1, 1, 10 and 100, and the OD_600_ of the latter was compared with that of the control (without phage) (bacterial host only); the bacterial load in the phage-treated group was greatly reduced. Even though mild fluctuations in bacterial growth were observed, it was maintained below 0.2 OD_600_ for all MOIs throughout the 5 h period.

### Bacteriophage insensitive mutant (BIM) frequency

Phage-resistant mutant generation was investigated at a higher MOI of 100 over periods of 16, 24, and 48 h. The results indicated the occurrence of phage-resistant mutants which were picked and re-spotted with ØPh_SS01 and confirmed the lack of plaques or clearance as depicted in Table 3.

**Table 2:** EOP of the alternative hosts that showed tropism to *S. saprophyticus*.

Phage	Host	Titre (PFU/mL)	EOP	SD
ØPh_SS01	*S. saprophyticus*	2.40E + 09	1.00E + 00	4.00E + 08
	*S. ureilyticus*	8.67E + 06	3.61E-03	1.15E + 06
	*S. epidermidis*	6.67E + 06	2.78E-03	1.15E + 06
	*S. arlettae*	9.33E + 06	3.89E-03	1.15E + 06
	*S. gallinarum*	9.33E + 06	3.89E-03	1.15E + 06
	*B. stratosphericus*	1.73E + 06	7.22E-04	1.78E + 08

**Table 3:** BIM frequency of *S. saprophyticus* over 16 h, 24 h and 48 h.

Bacterial host	Phage	BIM Frequency (CFU/mL)					
*S. saprophyticus*	ØPh_SS01	BIM 16 h	SD	BIM 24 h	SD	BIM 48 h	SD
		7.98E-06	4.494E-07	9.70E-06	5.46E-07	1.30E-05	2.50E-06

### Prophage induction assay

**Fig. 6:**
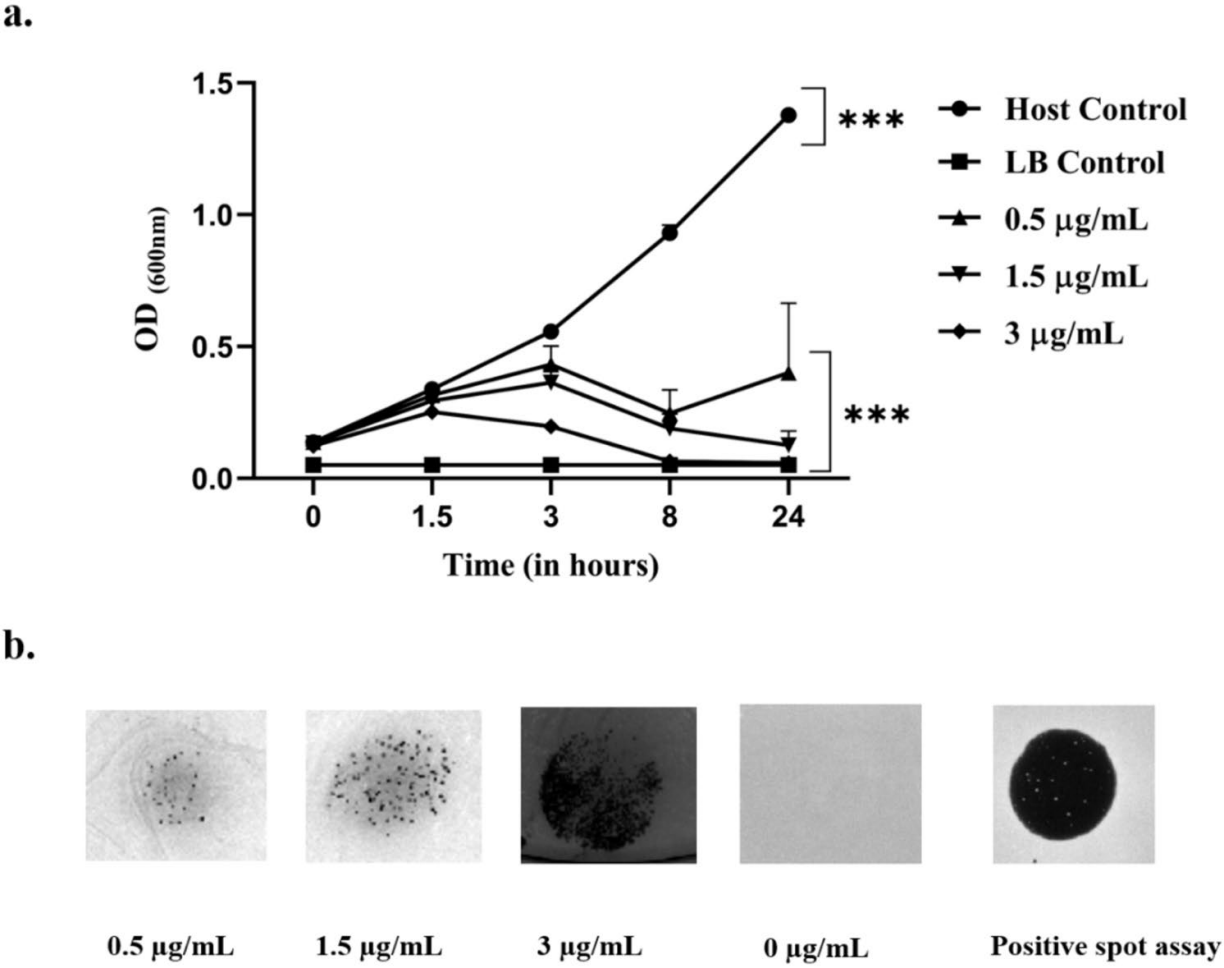
Induction of prophages from *S. saprophyticus* by mitomycin C. (**a**) Prophage induction assay of *S. saprophyticus* using mitomycin C over a period of 0, 1.5, 3, 8 and 24 h. (**b**) A spot assay of the effects of different concentrations of mitomycin C on the host bacterial lawn indicated a zone of hazy clearance, confirming the presence of prophages. Host bacterial growth decreased significantly with increasing concentrations of mitomycin C, ***: *p* < 0.001.

The results suggested the presence of prophage integration in *S. saprophyticus*, which was confirmed by the presence of turbid/hazy clearance after induction with mitomycin C. There was a significant reduction in the growth of the host bacteria on mitomycin C induction in comparison with the control (without mitomycin). Compared with the positive spot assay (known phage lysate), the turbid clearance indicated the presence of prophage associations, as shown in Fig. 6 (a, b) (Supplementary Figure S2).

### *S. saprophyticus* isolation and characterisation

Bacterial strains isolated from socks were subjected to biochemical characterisation followed by 16S rDNA ribotyping. The *S. saprophyticus* strain was identified to be gram-positive, coagulase-negative, urease-positive, and novobiocin-resistant.

### Phage-mediated reduction of *S. saprophyticus* in MS broth (liquid medium assay)

The reduction of *S. saprophyticus* via the MS broth liquid medium assay revealed the vital potential of phages as remedies for reducing the pathogen load. ØPh_SS01 was showing 100% efficacy over 24 h in comparison with the untreated control (control) as depicted in Supplementary Fig. S3(b) (*p *< 0.001). The treated set was checked for CFU/mL count, and they were able to identify ~ 7-log reduction. Compared with that of the control (C), treated (T), and blank (B), based on visual parameters, the colour of the MS broth drastically changed from pink to yellow in the control (phage untreated), whereas the blank and treated (phage treated) samples retained the typical pink colour, indicating the active growth of bacteria in the control, whereas the suppression of the same colour was observed in the treated, indicating reduced metabolism, as shown in Supplementary Fig. S3(a). The bacterial colony counts of the control and treated samples indicated surplus growth and growth inhibition, respectively, as shown in Supplementary Fig. S3(c).

### Biofilm Inhibition assay

**Fig. 7:**
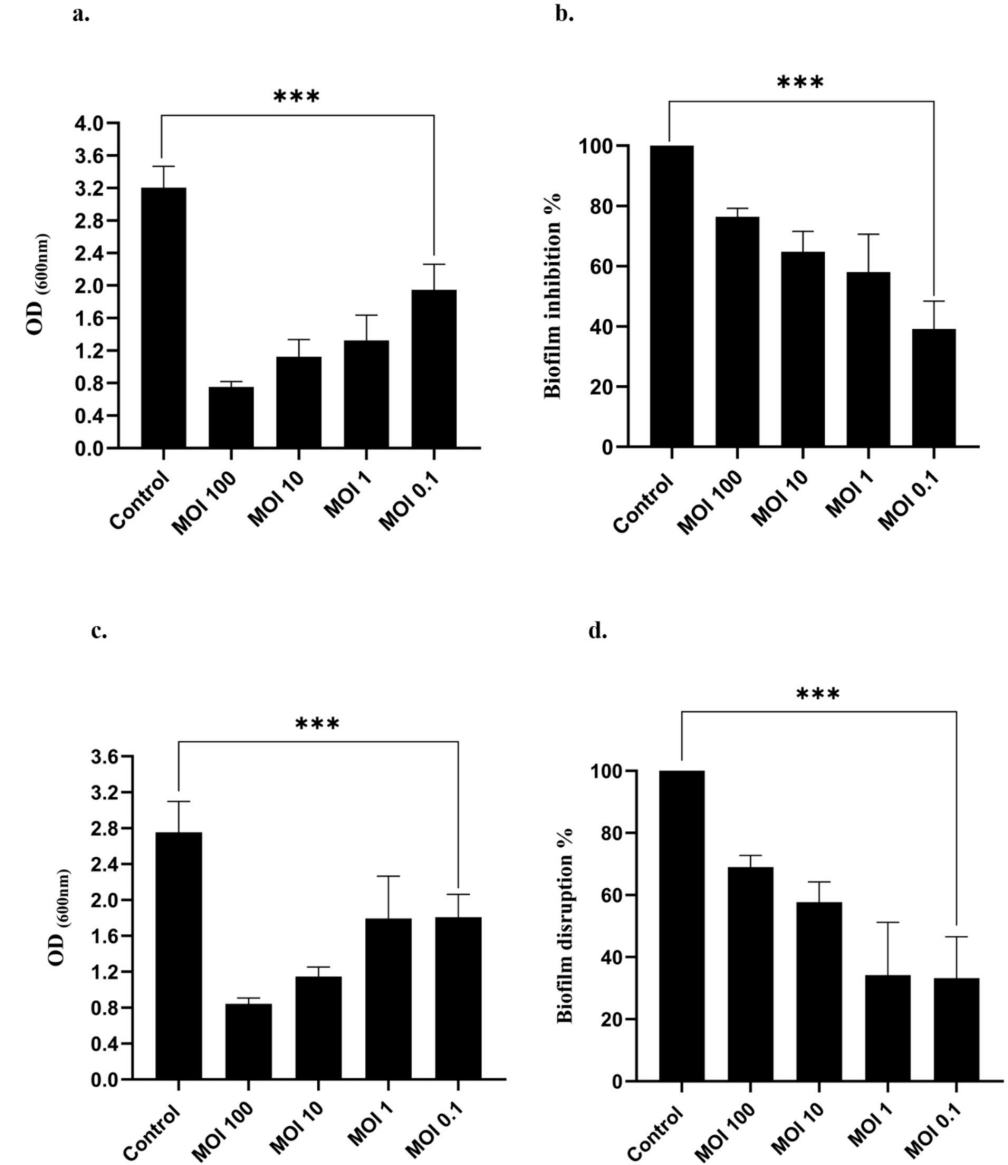
Biofilm inhibition and biofilm disruption of *S. saprophyticus.* (**a**) The biofilm inhibition efficacy of ØPh_SS01 at different MOIs was determined by calculating OD_600nm_. (**b**) The biofilm inhibition of ØPh_SS01 expressed in percentage; maximum activity at MOI 100 with 77 ± 2.9% biofilm reduction. (**c**) Biofilm disruption efficiency of ØPh_SS01 at varying MOIs identified by the optical density (OD_600nm_) method. (**d**) Percentage expression of biofilm disruptive capability of ØPh_SS01 with maximum efficacy of 69 ± 3.7% at MOI 100. Error bars represent standard deviation, and ***: *p* < 0.001 was considered significant.

**Fig. 8:**
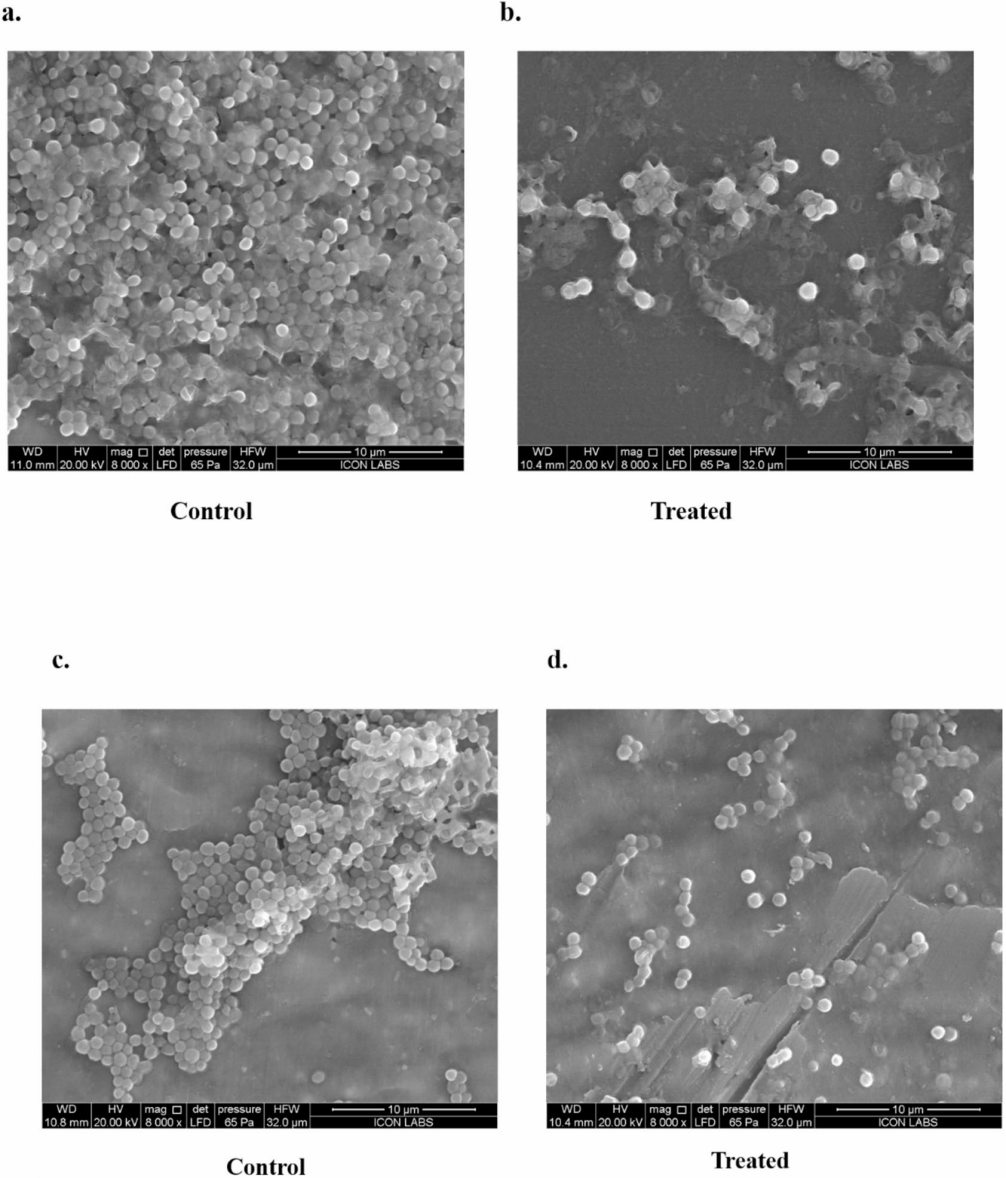
SEM (Scanning Electron Microscopy) imaging of ØPh_SS01 treated *S. saprophyticus* biofilm. (**a, b**) Biofilm inhibition capability of ØPh_SS01, control and treated comparison. (**c, d**) Comparison of control and treated biofilm by ØPh_SS01 (biofilm disruption).

*S. saprophyticus* and ØPh_SS01 were incubated together for approximately 18–24 h to identify the biofilm inhibition efficacy of the phage. Post-incubation after removal of planktonic cells, the biofilm formation of *S. saprophyticus* was assessed on comparing control (bacteria only) with treated (bacteria + phage). The biofilm inhibition efficiency of ØPh_SS01 at varying MOIs of 100, 10, 1, and 0.1 was determined to be 77 ± 2.9%, 65 ± 6.7%, 59 ± 12.5%, and 39 ± 9.2%, respectively, as depicted in Fig. 7(a, b). The SEM imaging of biofilm inhibition by ØPh_SS01 was also determined, as shown in Fig. 8(a, b).

### Biofilm disruption assay

The biofilm disruptive capability of *S. saprophyticus* was tested by treating ØPh_SS01 with an already formed biofilm of the bacteria. To decipher the efficacy of ØPh_SS01, control (only bacteria) and treated setups (with phage) were compared and analysed. The biofilm disruption efficiency of ØPh_SS01 was identified to be 69 ± 3.7%, 58 ± 6.4%, 38 ± 17%, and 41 ± 13.3% at different MOIs 100, 10, 1, and 0.1, respectively, as shown in Fig. 7(c, d). Biofilm disruption by ØPh_SS01 was subjected to SEM analysis as depicted in Fig. 8(c, d).

### Cytotoxicity testing against immortalized human keratinocytes cell lines (HaCat cells) and human urinary bladder carcinoma cell lines (T24 cell lines)

**Fig. 9:**
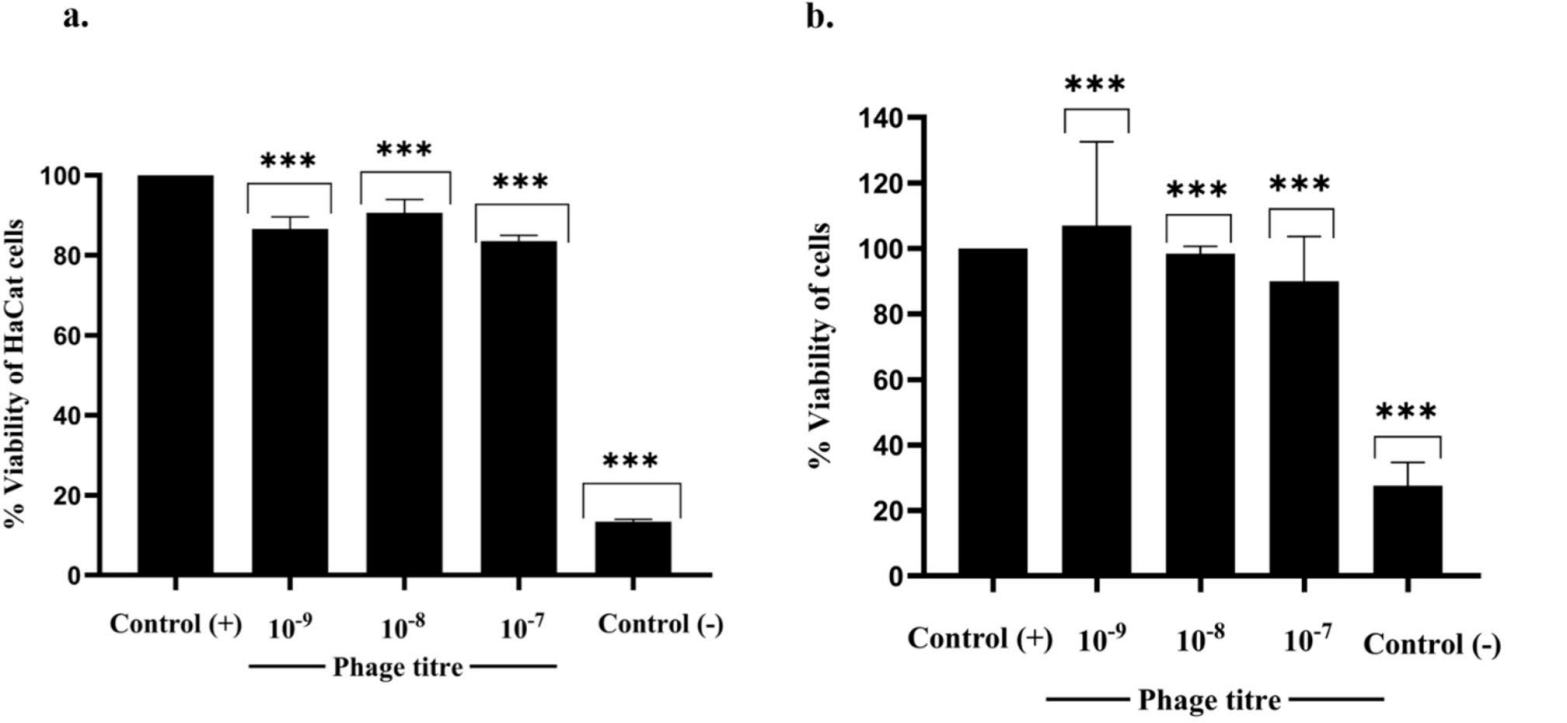
**(a,b)**Cytotoxicity of Ph_SS01 on host cell lines. The cytotoxicity of ØPh_SS01 on human keratinocytes and T24 lines was determined, where the control (+) was (DMEM + 10% FBS + cells), the treated setup had different phage dilutions (10^− 9^ to 10^− 7^), and the control (-) (0.1% Triton X) was maintained. On comparing with control, phage-treated cell lines showed a percentage viability of 87%, 91%, and 84% for each of the phage dilutions, whereas the viability of 0.1% Triton-X-treated cells was reduced to 13% for HaCat cell lines, while for T24 cells the percentage viability was 106%, 98% and 90% for each phage dilution, whereas in the case of 0.1% Triton-X the viability was reduced to 28% respectively, ***: *p* < 0.001.

The possible cytotoxicity of ØPh_SS01 on keratinocytes and T24 cell lines was determined by conducting an MTT assay. The ØPh_SS01 treated keratinocytes and T24 cells were given a media change and incubated at 37 °C in a CO₂ incubator (5% CO₂) for 24 h. Post-incubation the cells were again washed with fresh medium, treated with MTT (in dark conditions) and incubated for about 3–4 h in a CO₂ incubator. After incubation the media contents were aspirated, and the formazan crystals formed were dissolved in DMSO, and the optical density was quantified by measuring the absorbance at 570 nm. The viability of the control (untreated cells) was compared with treated (phage-treated), and the relative percentage of viability was estimated. Considering the control as 100%, the cells treated with different phage dilutions (10^− 9^ to 10^− 7^ PFU/mL) exhibited a minute reduction in viability (84 to 87%) and (90 to 106%), whereas the negative control (NC), i.e., 0.1% Triton X treated cell lines, exhibited a viability of 13% and 28% in HaCat and T24 lines respectively which evidently indicates the potential and non-toxicity of ØPh_SS01 as shown in Fig. 9(a, b).

### Potential application of phage in infection control by surface matrix immobilization

**Fig. 10:**
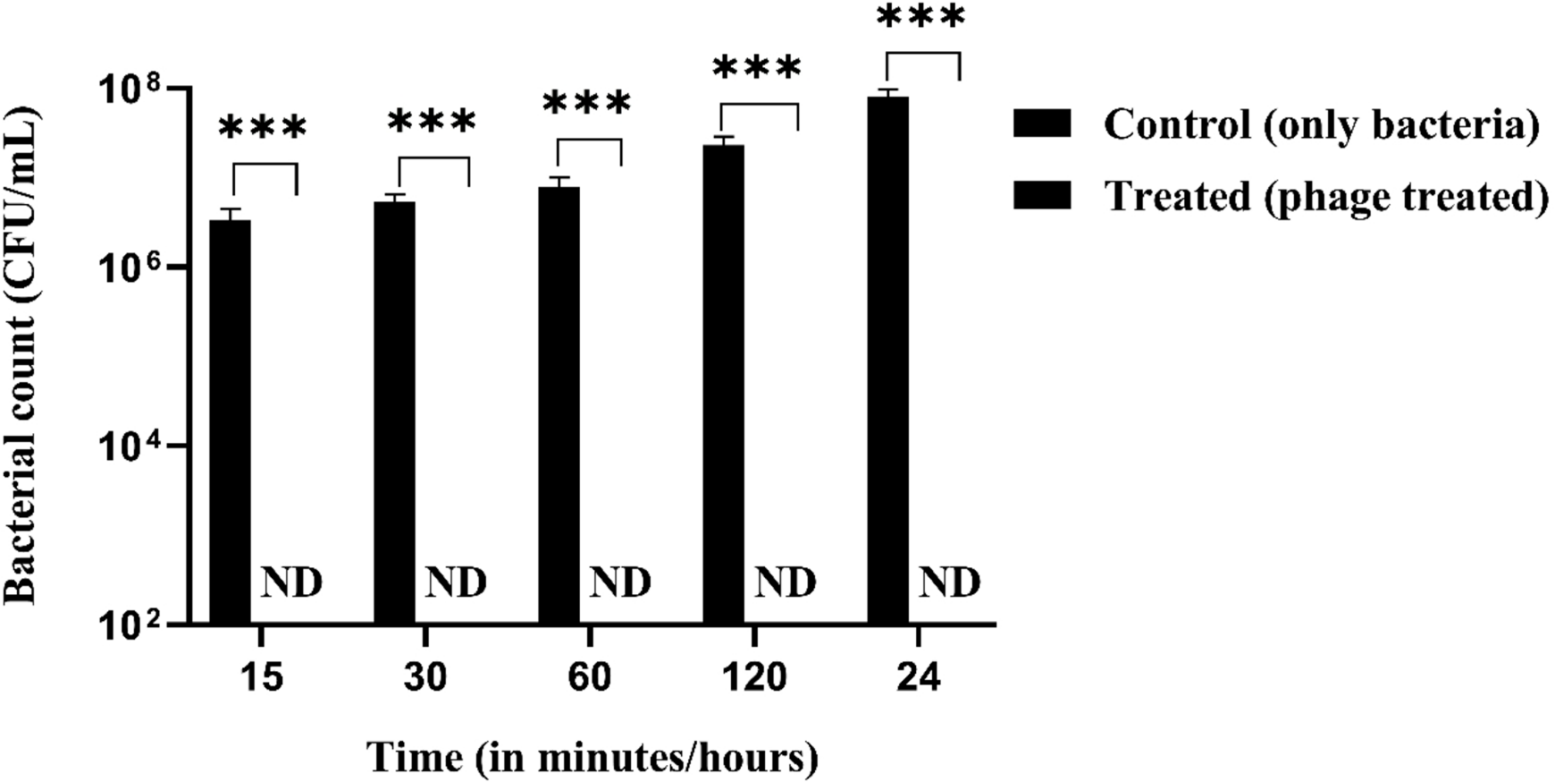
Phage immobilisation on textile matrices. The binding efficiency of phages on textile materials was analysed by comparing control (without phage) and treated (with phage) setups over a period from 15 min, 30 min, 60 min, 120 min and 24 h. Bacterial counts were not detected (ND) in the phage-treated setup indicating a 7-log reduction, ***: *p* < 0.001. ND, not detected.

The immobilisation of phages on different matrices gives rise to different application opportunities. Hence, we tested the binding efficacy of phages onto textile matrices. Commercially available cotton-based matrices were dipped with *S. saprophyticus* (0.2 OD_600_) and treated with ØPh_SS01 at an MOI of 100. On comparing the control (without phage treatment) with that of the treated (with phage), a 7-log reduction was observed over a period of 15 min, 30 min, 60 min, 120 min, and 24 h, as shown in Fig. 10. This could be explained as a proof of concept (POC) study for incorporating phages into textile matrices, which demonstrated their effectiveness in terms of bactericidal properties.

## Discussion

Bacteriophage-mediated mitigation of opportunistic infections and associated biofilms is an effective and translatable method, as phages tend to lyse bacteria substantially to a level below the infectious dose^27^. *S. saprophyticus*, a coagulase-negative *Staphylococcus* spp usually identified as a native commensal resident of the body responsible for imparting body odour, is recognised as the second most frequent causative agent of UTIs and associated biofilms in both male and female communities^28–30^. In the era of antimicrobial resistance, the generation of multidrug-resistant strains is an alarming and potent threat, so as foresight, we could attempt to control *S. saprophyticus*-associated in﻿fections and biogenic body malodour through effective application of bacteriophages, which have been reported only in very few instances so far^31,32^. Phages are generally very specific to the strains peculiar to space (geography) and time (climate). So far, phages against *S. saprophyticus* have not been reported from the Indian subcontinent. Since *S. saprophyticus-*mediated problems are opportunistic and cause secondary infections, ØPh_SS01 was employed in this study for establishing a proof of concept (POC) to control infection and biofilm formation. The efficacy of the phage ØPh_SS01 was determined through general characterisation, viz., morphological features (plaque size), TEM, tropism, adsorption efficiency, burst size, optimal MOI, stability, and bacteriolytic assay. Molecular characterisation was done by RFLP and WGS whereas *S. saprophyticus* was identified and characterised by novobiocin susceptibility, 16S rDNA ribotyping, antibiogram, BIM frequency, prophage induction, and EOP. The plaques produced by ØPh_SS01 were clear and pinpointed, indicating complete lysis of the bacteria^33^. TEM imaging revealed the phage was bearing a round head with long tails belonging to the class of *Caudoviricetes* (*Siphoviridae*)^34^. The phage was found to work most effectively at higher MOIs of 100 and 1000 with complete clearance, depicting the high efficacy and effectuality, thus deciphering the desired bacteriolytic effect. Hence, phages provide a promising insight to manage opportunistic and secondary infections. Also, it could be inculcated in promoting hygiene and sanitation, as it proves to be effective in controlling malodorous commensals.

The cross-reacting nature of phages is another attractive feature which enables them to infect multiple hosts, which could be interspecific or even intergeneric^35^. ØPh_SS01 exhibited extended polyvalency against several *Staphylococcal* spp including *S. arlettae*, *S. gallinarum*, *S. ureilyticus*, *S. epidermidis*, and *B. stratosphericus*. Polyvalent phages are highly effective in bacterial mitigation, as they can be utilised independently instead of formulating phage cocktails against a wide range of hosts and also eases the process of phage characterisation. Otherwise, detailed study on every single phage in the cocktail might increase redundancy. Another benefit accounts for the large-scale enrichment of phages using the surrogate or production host in substitution for the isolation host^36,37^. ØPh_SS01 hereby proves to be an effective tool for the reduction of *S. saprophyticus* and other coagulase-negative *Staphylococcus* spp (for POC establishment) responsible for various opportunistic/secondary infections and biogenic malodour. EOP was calculated for all the bacterial strains that showed tropism with ØPh_SS01, and it was identified that all of them have an EOP ratio below 1, proving them to be moderate production hosts^38^.

Stability of phages plays an important role in their storage, shelf life, and applications. The stability of phages over a wide range of pH, temperature, chloroform and saline concentrations was investigated. Phages for application in the human body should be able to withstand sweat and urine pH for effective bacterial control^39,40^. ØPh_SS01 was tested for its stability over a wide range of pH extending from strongly acidic to alkaline, portraying its high pH tolerance from 2 to 10, indicating its stability in human sweat and urine, which usually have pH ranges of 4.6–8 and 3.5–6.3, respectively^41^. The phage is also exhibiting extended thermal tolerance to 60 °C; stability up to 37–45 °C is sufficient for application in clinical and sanitary purposes and large-scale production of phages. Stability of phages in chloroform indicates the absence of lipids in their capsid proteins, provided they lack the enzyme machinery for lipid synthesis, and mostly those are host-driven; stability in organic solvents like chloroform indicates the absence of lipids^42^. ØPh_SS01 showed considerable stability up to 9% chloroform concentration without significant titre reduction, which could be utilised as an effective strategy for phage recovery in large-scale production instead of expensive methods like 0.22 μm membrane filters.

The adsorption efficacy of phages is considered relevant, as phages with an increased adsorption rate undergo enhanced bacterial lysis. About 91.5% of ØPh_SS01 was found to get adsorbed, releasing 67.08 ± 25 phage virions/cell which is considerably moderate in comparison to gram-negative bacterial phages. The size of the phages also affects the packaging of phage particles, resulting in a low burst size. Multiplicity of Infection (MOI) determines the number of phage particles available for bacterial infection in a particular scenario which barely interferes with the phage titre. The lytic to lysogenic shift cues are mostly estimated to be MOI^43^. The propensity of phages is usually determined by processing at a lower MOI, while ØPh_SS01 shows maximum lytic activity at a higher MOI (100)^44,45^. The infective nature of ØPh_SS01 excels at higher MOIs of 100, 10 and 1, while it has a minimised effect at lower MOIs of 0.1 and 0.001, the activity of the phage excels even at a very high MOI of 1000, 10,000 and 100,000. The phage’s activity was assessed over a 5-hour period, and it was found that all of the MOIs used were significantly reduced from 100 to 0.001 when compared to the host control.

The mitomycin C is used to induce prophages from the bacteria. Gram-positive bacteria harbour more prophages because of the expression of potential phage receptors by the thick peptidoglycan, which allocates more phage infection, leading to increased prophages from which bacteria engross vital metabolic traits^46,47^. Mitomycin C directly interferes with inhibition of DNA synthesis by guanine-cytosine cross-linking; at higher concentrations RNA and protein machinery are also halted. *S. saprophyticus*, when induced with mitomycin C, presented reduced growth and turbid clearance on spot assay, indicating the presence of prophage^48,49^. The occurrence of commensal opportunistic strains that are multi-drug resistant and the prevalence of antibiotic resistance are unavoidable. Harbouring and maintaining phages of these strains could effectively tackle the problem of emerging drug resistance^50^. *S. saprophyticus* demonstrated intrinsic resistance to novobiocin and exhibited resistance to all penicillinase-resistant beta-lactam antibiotics while remaining sensitive to other principal antibiotic groups. Raising phages against these strains could effectively manage infections and other complications without spurious use of antibiotics.

Phage resistance refers to bacteria acquiring resistance to previously sensitised phages^51^. Bacteriophage-insensitive mutants (BIM) were generated against *S. saprophyticus*, which, when rechecked with the sensitised phages, rendered inactive^52^. This could potentially affect the bacterial mitigation and require further novel phage preparations. The development of phage cocktails or polyvalent phage mixtures could prove effective to prevent resistance due to their broader efficacy, and the development of such mixtures depends on the detailed characterization of individual phages. Since *S. saprophyticus* phages remain underexplored and poorly studied, this work focuses on the isolation and comprehensive characterization of a single phage, providing essential groundwork for future cocktail formulations.

The liquid medium assay of ØPh_SS01 using MS broth to monitor *S. saprophyticus* reduction was highly significant, as there was 100% reduction over 24 h. On comparing the control and treated setups, the bacterial count was reduced considerably, proving it to be a better solution for controlling opportunistic infections. Biofilm inhibition and disruption efficiency of ØPh_SS01 was investigated to be 77% and 69%, respectively. The biofilm-disrupting properties could be effectively utilised in catheter-associated UTIs and other infections^53^. The non-cytotoxicity of the phage on human epidermal and T24 cell lines were proven by more than 85% viability of the cells and its yielding potential in therapy. The phage can also be potentially used for reduction of target bacterial loads associated with skin when delivered through textile materials on skin surfaces, sanitary napkins/pads and wound dressings^54^. The ability of phages to tolerate wide range of pH, and the efficacy with which it binds to different fabrics, could be leveraged for malodour mitigation and antisepsis^55^. Prevalence of *S. saprophyticus* as a commensal and its colonisation could lead to recurrent opportunistic infections, which can be alleviated using ØPh_SS01 in the form of formulations.

The genome sequencing of ØPh_SS01 revealed it as a double-stranded DNA phage with a sequence similarity of 91.57% with that of *Staphylococcus* phage TPWB-09 (Gen Bank ID : PQ358088.1). According to ICTV guidelines, a similarity of less than 95% can be considered as a novel species of the same genera (>50% similarity to be considered as a novel genus)^56^. The annotation revealed the presence of structural and regulatory proteins like head, neck, and tail proteins, along with proteins like holin and anti-repressor required for the lytic-to-lysogeny switch, clearly indicating the temperate lifestyle of the phage. Lytic phages are generally preferred over temperate ones for phage therapy, as temperate phages might mobilize virulence factors, toxin genes, or antibiotic resistance genes between bacteria. Thus, it might increase horizontal gene transfer and aid in the spread of antibiotic resistance. Lysogeny in bacteria increases chances of superinfection immunity, rendering subsequent phage infections ineffective^57^. While considering the dearth of phages against *S. saprophyticus*, ØPh_SS01 is an important resource and could be modified through specific gene deletion, homologous recombination, bacteriophage recombineering and CRISPR-Cas genome editing strategies to convert it to obligate lytic^58^. The proteome-based phylogenetic analysis also enabled to establish its novelty due to its seclusion in a separate clade amongst 36 related phages. Despite the temperate traits, its genomic attributes and stability indicate that, with suitable genetic alterations, it might be modified for specific lytic functions and practical uses in biotechnology.

## Conclusion

The ØPh_SS01 is a double-stranded DNA, temperate phage belonging to the class *Caudoviricetes.* It was isolated against coagulase-negative *S. saprophyticus and* showed stability across varying pH, temperature, and salt concentration. This novel phage could infect *S. saprophyticus* in its planktonic as well as biofilm lifestyles, thereby displaying its potential in mitigating opportunistic UTIs and other complications. Extended application for sanitation purposes can be considered another privilege. Thus, this study proves to be effective for controlling emerging problematic bacterial strains and declining indiscriminate antibiotic consumption.

## Materials and methods

### Buffers and media used for culturing

SM buffer (NaCl, 5.8 g/L; MgSO_4_, 2 g/L; 1 M Tris HCl, 50 mL/L; pH, 7.4) was used for storage and preparation of dilutions of the phage^59,60^. PBS buffer (NaCl, 80 g/L; Na_2_HPO_4_, 14.4 g/L; KCl, 2 g/L; KH_2_PO_4_, 2.4 g/L) was used for biofilm aspiration, washing and cell culture studies. Deca-strength (10X) bacteriophage enrichment broth (tryptic digest of beef heart, 10 g/L; NaCl, 5 g/L; dextrose, 1 g/L; and dipotassium hydrogen phosphate (K_2_HPO_4_), 80 g/L) was used for phage enrichment, which is a modified medium for isolation of phages against gram-positive bacteria. LB agar (Luria Bertani Agar, HiMedia Laboratories, India) and LB broth (HiMedia Laboratories, India) were used for routine culturing of the bacterial strains and for conducting all of the experiments. Phage purification and titre determination were done using the double agar overlay method (plaque assay). Owing to the minute size of the isolated phages, the concentration of agar (Hi Media Laboratories, India) (hard and soft agar) was reduced from 2% to 0.7% to 1.8% and 0.35%, respectively. Bacterial strains isolated were stored and maintained in Mannitol Salt Agar (MSA agar, HiMedia Laboratories, India). MS broth (protease peptone, 10 g/L; beef extract, 1 g/L; NaCl, 75 g/L; D-mannitol, 10 g/L; Tween 20, 10 mL/L; phenol red, 25 mg/L; pH, 7.4 ± 2) was used for establishing the POC (Proof Of Concept) of phage-mediated pathogen control. Cell culture studies were conducted primarily using HaCat cells (cryopreserved), other major materials used for cell line work were PBS buffer, Dulbecco’s Modified Eagle Medium (DMEM) supplemented with 10% foetal bovine serum (FBS), trypsin–EDTA solution, dimethyl Sulphoxide (DMSO), and MTT (3-(4,5-Dimethylthiazol-2-yl)-2,5-Diphenyltetrazolium bromide solution (Sigma Aldrich, USA).

### Isolation of bacterial strains and their culture conditions

All the bacterial strains used in the study are isolated from foetid sock samples sourced from anonymous individuals in Amrita Vishwa Vidyapeetham, Amritapuri Campus, Kollam, Kerala, India, and Unilever Industries Pvt Ltd, Bangalore, Karnataka, India. Samples from almost 50 individuals were obtained, from which 38 bacterial strains were isolated based on colony morphology and mannitol fermentation. The thirty-eight bacterial isolates were identified by 16S rDNA sequencing, and the predominant species identified were *Staphylococcus* spp. From the identified bacterial strains, *S. saprophyticus* was selected owing to its prevalence as a native commensal and its role in emerging and opportunistic UTI infections in both males and females. These strains were isolated in Mannitol Salt Agar (MSA agar, HiMedia Laboratories, India) to distinguish their fermentative property and were maintained in MSA agar as well as in Luria Bertani Agar (LB agar, HiMedia Laboratories, India) and LB broth (HiMedia Laboratories, India) for routine plating and culturing. The isolates were maintained thoroughly at 4 °C for the experimental proceedings. Glycerol stocks of all the isolated bacterial strains are maintained at -80 °C for extended storage.

### Antibiotic sensitivity test of *S. saprophyticus* and other strains used for tropism study - Kirby-Bauer disc diffusion method

Antibiotic sensitivity profiling of the bacterial host *S. saprophyticus* and other strains were carried out using major classes of antibiotics namely β-lactam antibiotics (Penicillin G - 2 units, Oxacillin-5 mcg, Methicillin-5 mcg, Amoxicillin-10 mcg, Ampicillin-10 mcg, Ticarcillin-75 mcg), carbapenems (Imipenem-10 mcg, Meropenem-10 mcg), third generation cephalosporins (Ceftazidime (CAC)-30/10 mcg, Ceftazidime (CAZ)-30 mcg), Tetracyclines (Minocycline-30 mcg), aminoglycosides (Gentamycin-10 mcg, Tobramycin-10 mcg, Amikacin-30 mcg), fluoroquinolone drugs (Ciprofloxacin-5 mcg, Levofloxacin-5 mcg), sulfonamide drugs (Co- Trimoxazole-25 mcg), macrolides (Erythromycin-15 mcg), polymyxins (Colistin-10 mcg) and aminocoumarin (Novobiocin-30 mcg) by Kirby-Bauer disc diffusion method. The respective bacterial strains were swabbed onto Muller-Hinton (MHA) agar plates following which antibiotic discs were dispensed into the plates and kept for overnight incubation at 37 °C. The zone of inhibition was measured and interpreted according to CLSI and EUCAST guidelines^61^.

### Bacteriophage enrichment and isolation

For enrichment and isolation of phages against *S. saprophyticus* wastewater samples were collected from the Effluent treatment plant (ETP), Amrita Vishwa Vidyapeetham, Amritapuri Campus, Kollam, Kerala, India, which is a centralised facility for effluent collection and treatment. Other sewage samples from the local regions of the institute, together with the ETP samples, were pooled for carrying out the phage isolation because of the challenges and difficulties in isolating phages against gram-positive bacteria in comparison with gram-negative strains. For enrichment of phages, 22.5 mL of sewage sample was mixed with 2.5 mL of deca-strength phage enrichment broth (modified), to which 500 µL of exponential/log phase (0.4 OD_600_) culture of the respective bacterial strains was added and allowed to incubate overnight at 37 °C in a shaking incubator (150–200 rpm). The resulting suspension was centrifuged at 7000 × g for 15 min at 4 °C and the supernatant was collected. The collected supernatant is expected to have susceptible phages and is subjected to filter sterilisation by passing it through a membrane filter (0.22 μm syringe filter, Thermo Scientific Nalgene, PES, 25 mm; China) for making the phage lysate devoid of bacterial contamination. Following the filter sterilisation, 5–10 µL of the lysate are spotted onto LB agar plates previously swabbed with susceptible host and are incubated at 37 °C overnight. The presence of phages was denoted by the zone of clearance observed in the respective bacterial plates, and it was confirmed by conducting a double agar overlay method (plaque assay)^60,64^.

### Phage purification and storage

The isolated phage lysates may be heterogenous in nature (presence of more than one type of phage for a single host), so to purify these phages, a plaque assay (double agar overlay method) was done for distinguishing them based on plaque morphology, in which 100 µL of crude lysate is added onto 900 µL of SM buffer and serially diluted up to 10^− 9^ dilutions. Each of these dilutions (10^− 1^ to 10^− 9^) was mixed with 100 µL of the respective host (*S. saprophyticus*) and incubated for 10–15 min; to that, 4–5 mL of 0.35% soft agar was added and poured into LB hard agar (1.8%) base. The agar concentrations of both hard agar and soft agar were reduced (1.8% and 0.35%) for enhanced visibility of the plaques owing to their minute size. The resulting plates are incubated at 37 °C overnight. The morphologically different plaques were separated by picking up the identical plaques and suspending them in 100 µL of SM buffer, followed by a plaque assay. The phage purification was done subsequently 4–5 times for each of the bacterial hosts to prevent the mixing of phages and to obtain a single pure phage lysate for further proceedings^65^. Phage titre determination was also carried out by serially diluting the pure lysate in SM buffer followed by spot assay. The phage titre is expressed as PFU/mL (plaque-forming units per millilitre). The phage lysates were maintained strictly at 4 °C during the entire experimentation, and glycerol stocks of the phage lysates were stored at -80 °C for long-time preservation.

### Transmission electron microscopy (TEM) analysis for morphological identification

TEM analysis was done using high-titre phage lysates (10^9^) using Tecnai G2 Spirit Biotwin (Transmission Electron Microscope) at 120 kV. The instrument is equipped with tungsten “W” filament and Olympus soft imaging solutions VELETA CCD (Tecnai imaging and analysis) with a magnification range of 35,000 X. The phage dimensions were obtained using ImageJ software. TEM analysis for the phage was processed from Icon Labs Pvt Ltd, Icon Sanpada, Navi, Mumbai, India.

### Scanning electron microscopy (SEM) for morphological assessment of biofilm reduction

SEM analysis was carried out to investigate the biofilm inhibition and disruption capability of ØPh_SS01. Biofilm formation was initiated in coverslips placed in 6-well plates for about 18–24 h, following which the planktonic cells were removed by 1X PBS wash in both control (only bacteria) and treated (bacteria with phage). The coverslips were then subjected to SEM analysis to identify the phage-mediated reduction of biofilm formed. SEM analysis was processed by Icon Labs Pvt Ltd, Icon Sanpada, Navi, Mumbai, India.

### Tropism (phage host range determination)

The tropism, or host range, of phages was determined against twelve gram-positive strains as described in Table 4. For determining the host range, 5–10 µL of the phage lysates were spotted onto LB plates previously lawned with the respective bacterial host. The phage lysate spotted plates were incubated at 37 °C overnight and observed for a zone of clearance. Clearance indicated the presence of cross-reacting phages, and it is further confirmed by serial dilution plating and plaque assays. The absence of tropism was indicated by the absence of clearance^66^.

**Table 4:** List of bacterial strains isolated from foetid socks for tropism establishment. The source and antibiotic resistance profile of all the strains used are depicted in the above table. Antibiotics used are penicillin G (P), Oxacillin (OX), methicillin (MET), amoxicillin (AMX), ampicillin (AMP), Ticarcillin (TI), ceftaxidime (CAC/CAZ), cefotaxime (CTX), Imipenem (IPM), meropenem (MRP), Ciprofloxacin (CIP), Levofloxacin (LE), gentamicin (HLG), tobramycin (TOB), Amikacin (AK), erythromycin (E), Minocycline (MI), colistin (CL), Co-Trimoxazole (COT) and Novobiocin (NV).

Sl. no	Bacteria	Source	Accession number/strain designation	Antibiotic resistance profile
**Gram-Positive bacteria**				
1	*Staphylococcus saprophyticus*	Socks	PV355097	NV, CIP, CAZ, TI, AMP, AMX, MET, OX, P
2	*Staphylococcus gallinarum*	Socks	PV211287	P, MET, AMX, AMP, CAZ, TI, LE, AK, TOB
3	*Staphylococcus arlettae*	Socks	PV195003	P, MET, AMX, AMP, CAC, TI, E
4	*Staphylococcus ureilyticus*	Socks	PV126630	P, MET, AMX, AMP, TI, E
5	*Staphylococcus warnerii*	Socks	PV124195	P, MET, AMX, AMP, CAC, TI, E, TOB
6	*Staphylococcus pasteuri*	Socks	PV168561	P, MET, AMX, AMP, CAZ, CAC, TI
7	*Staphylococcus haemolyticus*	Socks	PV188579	P, MET, AMX, AMP, TI, LE
8	*Staphylococcus epidermidis*	Socks	PV211443	P, MET, AMX, AMP, CAC, TI, CIP, LE, TOB
9	*Staphylococcus aureus*	Clinical	MRSA	P, MET, AMX, AMP, TI, CIP, LE, TOB
10	*Bacillus stratosphericus*	Socks	PV211303	P, MET, AMX, AMP, CAZ, CAC, TI, LE
11	*Bacillus safensis*	Socks	PV211323	P, MET, AMX, AMP, CAZ, IPM, AK
12	*Staphylococcus epidermidis*	ATCC	12,225	P, MET, AMX, AMP, CAC, TI, LE, AK

### Stability of phages at different temperatures and pH

The stability of the phage at different temperatures and pH was determined. Temperature conditions ranging from 4 °C, 20 °C, 37 °C, 45 °C, 50 °C and 60 °C were employed for ØPh_SS01; the temperature range was extended up to 70 °C for determining enhanced tolerance. For assessing the temperature stability of the phages, the lysates were incubated at the susceptible temperature for 1 h, followed by sequential serial dilution in SM buffer after cooling to room temperature. The dilutions were spotted onto LB plates swabbed with the respective bacterial host, and the titre of the phage was determined in PFU/mL^67^. Similarly, to determine the pH stability, the phage lysate was subjected to varying pH ranging from 2, 4, 6, 7, 8, and 10. The lysates were serially diluted in pH-adjusted SM buffer, and the resulting mixture was incubated at 37 °C for 1 h. The dilutions were then spotted on LB plates previously swabbed with the respective host to determine the phage titre^68^.

### Stability of phages in organic solvents (chloroform)

The stability of phages in chloroform indicates phages with simple capsid structure and a lack of lipid membranes. The chloroform stability of phages was determined by mixing equal volumes of phage lysate (500 µL) and respective chloroform dilutions (500 µL) ranging from 1%, 3%, 5%, 7%, and 9%, respectively^69^. The resulting mixture was incubated for 20–30 min at 37 °C in a shaking incubator (150–200 rpm). After incubation the resulting mixtures were centrifuged at 7000 × g for 15 min at 4 °C and the supernatant was serially diluted in SM buffer. The titre determination of the phages was carried out by conducting a spot assay in which the dilutions were spotted on LB plates previously lawned with the respective bacterial culture. Since chloroform is lethal for bacterial growth, it could be used as an effective strategy for phage lysate purification rather than using expensive methods like 0.22 μm syringe filtration^70^.

### Stability of phages in saline

The stability of phages at varying salt concentrations was investigated to analyse its solidity in surviving and resisting the DNA and protein denaturation and also its binding efficacy. The experiment was preceded by incubating 100 µL of phage lysate (10^8^ PFU/mL) and 900 µL of saline concentrations ranging from 0.5%, 1.5%, 3.5%, 5.5%, 7.5%, 9.5%, 12.5% and 20%, respectively. The respective phage saline mixtures were incubated for 1 h at 37 °C, after which they were serially diluted in corresponding saline solutions. The dilutions were spotted onto LB plates previously lawned with *S. saprophyticus*, and the phage titre was determined to estimate the stability^71^.

### Optimal MOI determination

The optimal ratio of bacteriophages to that of the bacterial host that would yield the highest progenies/virions per bacterial cell accounts for the optimal MOI. For this experiment phages and bacterial hosts were mixed at different MOIs 0.001, 0.01, 0.1, 1, 10, 100, 1000, 10,000 and 100,000 were incubated at 37 °C in a shaking incubator (150–200 rpm) for 5 h. The resulting mixture was then centrifuged at 7000 × g for 15 min and the supernatant was carefully drawn and serially diluted in SM buffer for phage titre determination. Based on the phage titre the optimal phage-to-bacterial ratio was determined. The titre determination was carried out by spot assay^72^.

### Phage adsorption assay

The time required for the phages to bind to the bacterial surfaces and initiate infection could be accounted for as the adsorption time^73^. Here, the percentage of unadsorbed phages was determined for validation of the time required for adsorption. For this experiment bacterial culture (4 mL) adjusted to 0.3 OD_600_ (10^6^ CFU/mL) was mixed with susceptible phage (1 mL- 10^6^ PFU/mL) thereby achieving an MOI of 1. The resulting mixture was incubated at 37 °C in an incubator, and 300 µL of the sample was drawn every consecutive 2 min starting from 0 to 14 min. The drawn samples were strictly kept in ice, and after the incubation period, all of the samples were centrifuged at 7000 × g for 10–15 min (4 °C). The supernatant from each time interval was collected and serially diluted in SM buffer to obtain the titre of the unbounded or unadsorbed phages and was determined by plaque assay.

### One-step growth curve and burst size calculation

The number of progeny phages or virions released per bacterial host cell accounts for the burst size^74^. For determining burst size, 1 mL of the respective bacterial host was adjusted to 0.3–0.4 OD_600_ (10^6^ CFU/mL), followed by centrifugation of the bacterial culture at 13,000 × g for 15 min at 4 °C. The resultant bacterial pellet was washed 2–3 times in SM buffer and finally resuspended in 1 mL SM buffer. 900 µL of the SM buffer constituted culture, and 100 µL of the susceptible phage (10^5^ PFU/mL) was mixed to attain an MOI of 0.1. The phage-bacteria mixture was incubated for 15–20 min at 37 °C in a dry bath, followed by centrifugation at 13,000 × g for 15 min at 28 °C. The resultant pellet was resuspended in 1 mL LB broth, and it was serially diluted up to 10^− 3^ in 9 mL LB broth. The dilutions were incubated at 37 °C (150–200 rpm), and 100 µL aliquots were drawn every 10 min starting from 0 min up to 100 min and each of the aliquots was mixed with 100 µL of the respective bacterial hosts along with 3–4 mL of 0.35% soft agar and a plaque assay was carried out. Burst size was calculated as the ratio of the average virions present in the latent period to the average of virions released during the rise period. The experiment was done independently with triplicates in each experiment^75^.

### Bacteriolytic assay

The bactericidal effect of phage against the host was determined by a bacteriolytic assay conducted in a 96-well microtiter plate (Thermo Scientific, Denmark). 0.1 mL of each bacterial culture (~ 10^6^ CFU/mL) was mixed with 0.1 mL of susceptible phages (~ 10^8^ PFU/mL) at different MOIs of 0.01, 0.1, 1, 10, and 100 respectively and were incubated at 37 °C in a rotary shaker at 150–200 rpm^76^. *S. saprophyticus* without phages and LB broth without bacteria were used as positive and negative controls, respectively. SM buffer served as a blank. The 96-well plate subjected to incubation was read at every consecutive 1 h starting from 0, 1, 2, 3, 4 upto 5 h. The phage-mediated bacteriolytic/bactericidal activity was measured at 600 nm in a microplate reader (Bio Tek, USA).

### Mitomycin C induction (prophage) assay

The presence of prophages in gram-positive bacteria could be identified by mitomycin C induction. *S. saprophyticus* was subjected to prophage induction, for which 200 µL of bacterial culture in logarithmic phase 0.2 OD_600_ was mixed with mitomycin C (10 mg, Merck, USA) at different concentrations ranging from 0, 0.5, 1.3, and 3 µg/mL, respectively. Bacterial culture devoid of mitomycin C was taken as a control, and LB broth served as blank. The resulting mixtures were subjected to incubation at 37 °C in a shaking incubator at 150–200 rpm, and the bacterial growth at different concentrations of mitomycin was measured (OD_600_) at 0, 1.5, 3, 8 and 24 h, respectively. To confirm the presence of prophages, the bacterial cultures with varying mitomycin concentrations were centrifuged at 7000 × g for 5 to 10 min. The resulting supernatant was collected and spotted onto LB agar plates which were priorly lawned with the respective host^77^.

#### Phage DNA isolation by phenol – chloroform method

The phage DNA isolation was carried out using the phenol-chloroform method^78^. This protocol proceeds with a three-day incubation cycle. For this, 800 µL of high-titre phage lysate was mixed with 200 µL PEG-NaCl (PEG 8000, 200 g/L; 2.5 M NaCl, 150 g/L) and incubated at 4 °C overnight for the precipitation of phages. The resulting mixture was centrifuged at 18,000 × g for 30 min at 4 °C and the pellet was collected and resuspended in 500 µL 5 mM MgSO₄.The samples were further treated with 1.25 µL of DNase (1000 U, Thermo Fisher Scientific, Lithuania) along with 2.50 µL of RNase I (10 mg/ml, Thermo Fisher Scientific, Lithuania) and were incubated at 37 °C for 1 h without shaking in a dry bath. Post incubation it was further treated with 1.25 µL of proteinase K (20 mg/mL Thermo Fisher Scientific, Lithuania), 25 µL of 10% SDS and 20 µL of 0.5 M EDTA mix well and incubated at 60 °C for 1 h. Post incubation, allow the tubes to cool and add an equal volume of phenol: chloroform (1:1) and invert the tubes several times for even distribution of the contents, followed by centrifugation of the mixture at 3000 × g for 5 min at room temperature. The supernatant (upper clear solution) was transferred carefully, and again the phenol: chloroform step was repeated. The transferred aqueous supernatant was added with an equal volume of chloroform and thoroughly mixed, followed by centrifugation at 3000 × g for 5 min. The resulting supernatant was collected, and 50 µL of 3 M Sodium acetate (NaOAc.3H_2_O) and 2.5 volumes of ice-cold absolute ethanol were added. The mixture is mixed well and incubated at -20 °C overnight. Following day for DNA precipitation, the sample was centrifuged at 10,000 × g for 20 min, and the supernatant was discarded. Subsequently, the tubes were half-filled with 1 mL of 70% ethanol and spun at 10,000 × g for 2 min, and the same step was repeated twice, and finally remove the ethanol. Leave the tubes open for about 15–30 min for the excess ethanol to vaporise. Finally dissolve the pellets in molecular-grade water. The DNA quantity and quality were determined by conducting an agarose gel electrophoresis in 1% agarose (HiMedia Laboratories, India) incorporated with ethidium bromide (10 mg/mL, Sigma Aldrich, USA). The isolated DNA sample was quantified using a Nanodrop spectrophotometer (Thermo Fisher Nanodrop 2000).

### Restriction fragment length polymorphism (RFLP) analysis

This technique was employed to identify the unique cutting pattern of the phage DNA by different restriction endonucleases. The isolated phage DNA was subjected to restriction digestion by enzymes like *Eco*RI, *Bam*HI, *Hind*III (Takara, Japan), *SSp*I, *Kpn*I, *PmI*I, *Hpa*I (Thermo Fisher Scientific, Lithuania) and phage DNA without restriction enzymes was kept as a control (uncut). The digestion conditions and durations were followed according to the manufacturer’s directions. The digested phage DNA bands after the suggested incubation were run on 1.2% agarose gel (HiMedia Laboratories, India) with ethidium bromide (10 mg/mL, Sigma Aldrich, USA). The restriction pattern of the digested fragments was visualised using a Nanodrop spectrophotometer (Thermo Fisher Nanodrop 2000)^79–81^.

### Whole genome sequencing, assembly, annotation and phylogenetic analysis

The bacteriophage DNA was isolated using the phenol-chloroform method and was subjected to Whole genome Sequencing (WGS) at Genotypic Technology Pvt Ltd, Bangalore, India, using Nanopore PromethION sequencer^82^. The raw data QC was performed using NanoStat-v1.6.0^83^. The adapter trimming of the raw data was done using Porechop-v0.2.4. De novo assembly was carried out for processed long reads using the Flye-v2.9.5 tool^84^. The gene/protein prediction was carried out for the draft genome using Prokka-v1.14^85^. The predicted proteins were further annotated using DIAMOND-v2.1.9^86^. The genome annotation was done using the PHAStEST tool and Proksee tool was adopted for visual representation of the phage genome. The phage lifestyle detection was carried out with the PhaBOX8-v2.1.12 tool^87^. The similarity of the phage with other closely related phages was identified by BLASTn analysis in NCBI. For determining the evolutionary relationships and taxonomic classification among the different phages available in the database, a proteome-based tree was established using a VipTree platform^88^.

### Bacteriophage-insensitive mutants (BIM) frequency

The bacteriophage-insensitive mutants of the host strain were identified. For this, 100 µL of the bacterial strain at 0.3 OD_600_ was mixed with 100 µL of phage lysate, and the mixture was incubated for about 10–15 min at 37 °C at an MOI of 100. After incubation the respective phage-bacterial sets were added with 4–5 mL of 0.35% soft agar and poured onto 1.8% hard agar. The plaque assay plates were subjected to further incubation at 37 °C for 16, 24, and 48 h. The BIMs that appeared after the respective time period were picked, and the ratio of the surviving colonies to that of the initial number of bacteria was identified to be the BIM frequency^89^. To guarantee phage resistance, the BIM colonies were streaked separately and subsequently spotted with phage lysate to confirm resistance.

### Efficiency of plating (EOP)

The efficiency of the phage to cross-react and infect other sensitive bacterial strains was determined^90^. The phage was serially diluted in SM buffer, and 10 µL of the dilutions were spotted onto LB plates previously lawned with sensitive hosts (as depicted in Table 1). After overnight incubation at 37 °C, all of the incubated plates were examined for obtaining EOP. The ratio of average PFU/mL of the secondary host to that of the average PFU/mL of the original host could be attributed as EOP^91^. Efficiency of plating could be indicated as the efficacy of the bacterial strains to be utilised as effective production hosts. Higher EOP indicates high efficacy to be used as a production host. The average EOP of the respective phage and bacteria combination could be attributed as high, medium, low and inefficient based on the PFU/mL ratio^36^.

### *S. saprophyticus* reduction using ØPh_SS01 in MS broth

The ability of phages to effectively reduce bacterial growth can be investigated using MS broth (beef extract, 1 g/L; NaCl, 75 g/L; protease peptone, 10 g/L; D-mannitol, 10 g/L; Tween 20, 10 mL/L; phenol red, 25 mg/L; pH, 7.4 ± 2). The high salt concentration of MS broth limits the growth of bacterial spp to *Staphylococcus.* Three setups were established as per need; MS broth itself served as a blank. Control setup was MS broth spiked with bacteria, SM buffer and 10X TSB broth, while the treated setup was inoculated with bacteria and phage. The reduction of bacterial growth was measured every 1, 3, 6, 9, 12 and 24 h and expressed in CFU/mL by comparing control and test.

### Biofilm Inhibition assay

*S. saprophyticus* requires about 18–24 h for biofilm formation and was investigated using microtitre plates (Thermo Scientific, Denmark)^92^. The respective host was inoculated in LB broth, and growth was adjusted to 0.2 to 0.3 OD_600_. 100 µL of *S. saprophyticus* was added onto control and treated slots in the 96-well plates (Thermo Scientific, Denmark). 100 µL of phage was added onto the treated, while 50 µL of SM buffer and 50 µL of 10X TSB were added onto the control. LB served as a negative control, and bacteria alone was maintained as a positive control. The plate was incubated at 37 °C for about 18–24 h. After the incubation, planktonic cells from the well plates were aspirated gently with the pipette and were washed 2–3 times with PBS buffer. Then the wells were flooded with 200 µL of 1% Crystal violet (CV) dye and incubated at 37 °C for 20–30 min. The stain was aspirated post-incubation and again washed 2–3 times with PBS buffer. Finally, 200 µL of 33% acetic acid was added, and the absorbance was measured at 575–600 nm in a microplate reader (BioTek, USA)^93^.

### Biofilm disruption assay

Biofilm formed by *S. saprophyticus* could be disrupted using bacteriophages even after colonisation^94^. This could also be investigated using microtitre plates (Thermo Scientific, Denmark). 100 µL of bacteria was added to control and treated slots of the microtitre plates and incubated for 18–24 h at 37 °C. Post-incubation control was added with 50 µL of SM buffer and 50 µL of 10X TSB and the treated slot was added with 100 µL of phage lysate. The well plate was again subjected to 3–5 h of incubation at 37 °C. The planktonic contents were aspirated and washed 2–3 times with PBS buffer. 200 µL of crystal violet was added to all the control and test wells and incubated at 37 °C for 20–30 min. Stain was aspirated and washed 2–3 times with PBS buffer, and 33% acetic acid was added to the wells. The absorbance was measured at 575–600 nm in a microplate reader (BioTek, USA).

### Cytotoxicity testing of ØPh_SS01 against epidermal cell lines (HaCat cells) and urethral cell lines (T24 lines)

The cellular toxicity of phages was evaluated to prove the potential of phages as an effective means of infection control^95^. The first step was to revive HaCat cells (immortalized human keratinocytes) and T24 (human urinary bladder carcinoma cell lines) frozen in liquid nitrogen and it was transferred into T25 flask containing DMEM (Thermo Fisher Scientific, Denmark) supplemented with 10% FBS (Thermo Fisher Scientific, Denmark) and incubated at 37 °C for 24 h in (5% CO_2_) CO_2_ incubator (New Brunswick™ CO_2_ Incubator, Germany). Following incubation, the cells were subjected to microscopic observation to ensure confluency and avoid contamination, if the cells had attained the required confluency, the media was discarded and treated with 500 µL of trypsin to detach the cells for 5 min and incubated at 37 °C for 24 h. The cells could be passaged for two or three generations in a similar way for obtaining healthy vital cells. When required confluency was obtained, the cells were treated with 10 µL of ØPh_SS01 by aspirating the media from the wells and again reconstituting 90 µL of DMEM (Dulbecco’s Modified Eagle medium) with 10% FBS (Foetal Bovine Serum) followed by incubation at 37 °C for 24 h in a CO _2_ incubator. Post incubation, the media was aspirated from the wells and again constituted with 90 µL DMEM with 10% FBS followed by the addition of 10 µL MTT in the control, blank, positive control (Triton X treated), as well as phage-treated wells in dark conditions (owing to its light sensitivity), succeeded by 4 h incubation in a CO _2_ incubator. The metabolically active cells convert MTT to formazan crystals, which were dissolved in 100 µL of DMSO and absorbance was measured at 570 nm in a microplate reader (Bio Tek, USA).

### Phage immobilization in textile materials

The ability of phages to bind onto textile materials could be a promising application in clinical and sanitary settings^96^. It could be extended to sanitary napkins, wound dressings and so on^97^. For this study we have used autoclaved 1 cm × 1 cm textile pieces (cotton socks from Adidas India Private Limited). *S. saprophyticus* culture was adjusted to 0.2 OD_600_ (10^6^ CFU/mL), and 1 mL of the same was added to textile pieces in the control (only bacteria) and treated (bacteria + phage) setup, followed by 15 min incubation at 37 °C and sequential drying. Post incubation treated setup was added with 1 mL phage lysate, and the control was added with 500 µL SM buffer and 500 µL 10 X TSB broth and then incubated at 37 °C for 15 min, 30 min, 60 min, 120 min, and 24 h in an incubator. Textile pieces after incubation were transferred to fresh tubes to ensure removal of residual phages, and then the control and treated textile pieces were immersed in SM buffer and serially diluted to determine the CFU/mL count, indicating the efficacy of phages to bind onto textile matrices and help in reducing bacterial infections. The MOI was maintained as 100 to ensure maximum binding and activity.

### Statistical analysis

GraphPad Prism 8.0 (GraphPad Software Inc., San Diego, CA) was used for analysing statistical significance. The stability studies, such as pH and temperature, were analysed using ordinary one-way ANOVA followed by Dunnett’s multiple comparison test with a single pooled variance whereas optimal MOI, salt concentration and chloroform stability was employed Tukey’s multiple comparison test. Bacteriolytic assay was analysed by two-way ANOVA followed by Tukey’s post-hoc test, and prophage induction studies by two-way ANOVA followed by Dunnett’s test. Liquid medium assay analysis was conducted using two-way ANOVA followed by Tukey’s post-hoc test with a single pooled variance. Biofilm inhibition and disruption were employed ordinary one-way ANOVA followed by Dunnett’s multiple comparison test with a single pooled variance. Phage immobilization study was analysed using a two-way ANOVA followed by Tukey’s multiple comparison test. Cytotoxicity of phage on HaCat and T24 cells was employed with one-way ANOVA followed by Tukey’s test. The significance was considered at *p* < 0.05, and all data expressed in mean values from three independent experiments ± standard deviation.

## Supplementary Information

Below is the link to the electronic supplementary material.

Supplementary Material 1

## Data Availability

The 16S rDNA sequences of *the strains* used in this study are deposited in GenBank ( [https://submit.ncbi.nlm.nih.gov/subs/genbank/](https:/submit.ncbi.nlm.nih.gov/subs/genbank) ) with accession numbers indicated in Table 4. The whole-genome sequencing of ØPh_SS01 was deposited at GenBank under the Bioproject accession number PRJNA1330512.
