# Supplementary material for: Isolation, characterisation and potential applications of a novel bacteriophage targeting beta-lactam-resistant Staphylococcus saprophyticus
Source: Sci Rep. 2026 Feb 5;16:7460. doi: 10.1038/s41598-026-35899-3 (PMC12929604; doi:10.1038/s41598-026-35899-3)
Supplement: Supplementary file 1 — Supplementary Material 1 [file 41598_2026_35899_MOESM1_ESM.docx]

**Isolation, characterisation and potential applications of a novel bacteriophage targeting beta-lactam-resistant *Staphylococcus saprophyticus***

**Gopika O^1^, Niti Sarat^1^, Maanya Manikandan^1^, Sumana S^1^, Parvathi Mohanan P C, Ajith Madhavan^1^*, Sandeep Varma R^2^, Samiran Mahapatra^2^, Bipin G Nair^1^, Sanjay Pal^1^***

^1^Amrita Vishwa Vidyapeetham. Kollam, Kerala. PIN- 690525, India

^2^ Unilever R&D Bangalore; 64, Main Road, Whitefield, Bangalore 560066, India

*Corresponding authors - Email ID: [sanjaypal@am.amrita.edu](mailto:sanjaypal@am.amrita.edu) (S. Pal)

[ajithm@am.amrita.edu](mailto:ajithm@am.amrita.edu) (A. Madhavan)


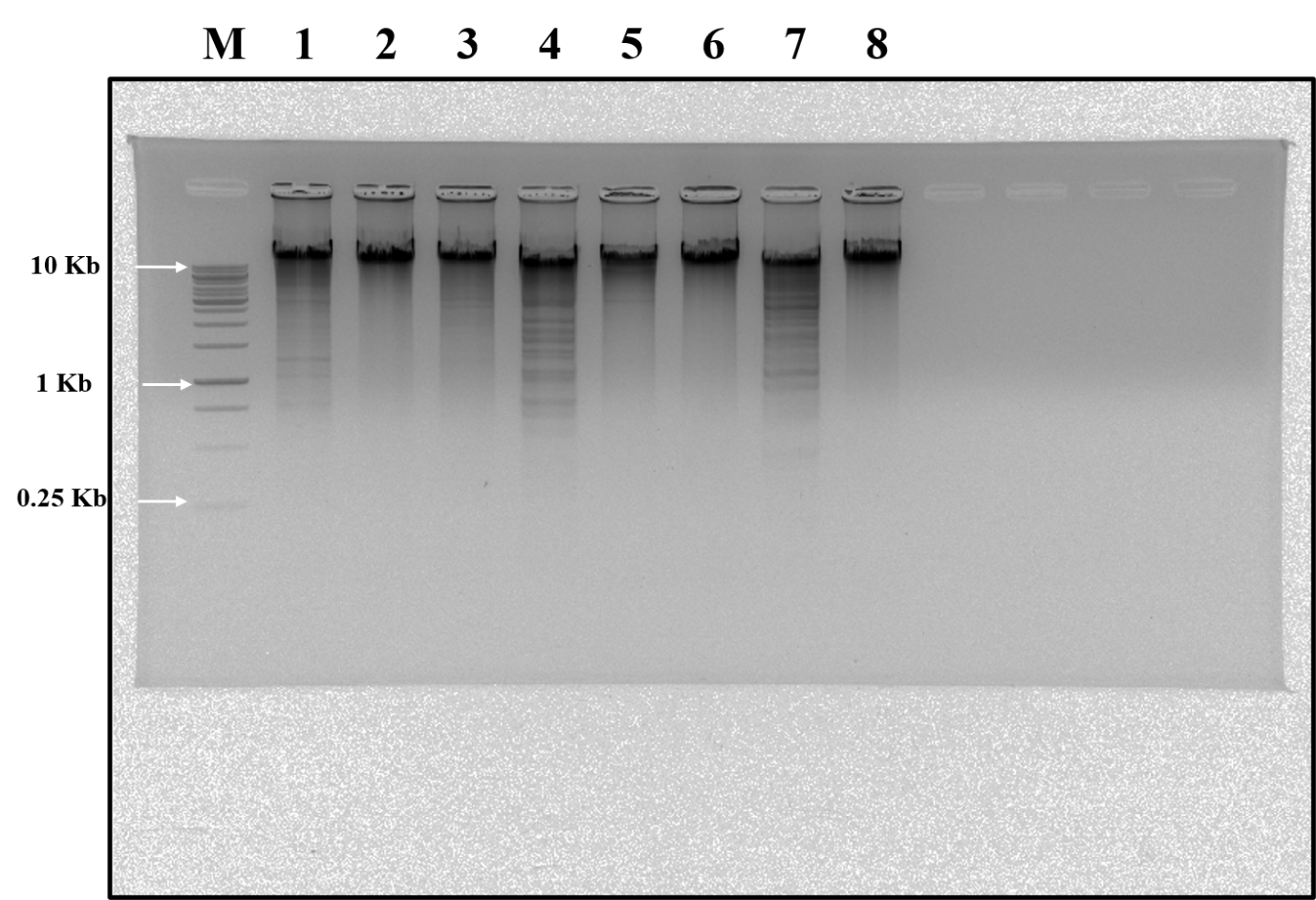


**Figure S1**. Restriction digestion profiling of ØPh_SS01 on 1.2% agarose gel with ethidium bromide. ØPh_SS01 subjected to restriction digestion; M – 1Kb DNA ladder (Gene ruler, Thermo Scientific), lane 1 – *Eco*RI, lane 2 – *Bam*HI, lane 3 – *Hind*III, lane 4 – *Ssp*I, lane 5 – *Kpn*I, lane 6 – *PmI*I, lane 7 – *Hpa*I, lane 8 - Uncut Phage DNA.


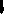


| Antibiotic Classes | Antibiotics | Zone of inhibition in mm | Sensitivity profile | Zone of inhibition in mm (*EUCAST* guidelines) |
| --- | --- | --- | --- | --- |
| Beta-lactams | Penicillin G | 17 | R | ≥26 |
|  | Oxacillin | 0 | R | ≥18 |
|  | Methicillin | 0 | R | ≥17 |
|  | Amoxicillin | 13 | R | ≥28 |
|  | Ampicillin | 0 | R | 18 |
|  | Ticarcillin | 21 | R | ≥23 |
| 3rd generation cephalosporins | Ceftaxidime | 13 | R | ≥14 |
|  | Cefotaxime | 23 | S | 23 |
| Carbapenems | Imipenem | 25 | S | ≥28 |
|  | Meropenem | 24 | S | ≥16 |
| Fluoroquinolones | Ciprofloxacin | 23 | R | ≥24 |
|  | Levofloxacin | 19 | S | 19 |
| Aminoglycosides | Gentamicin | 24 | S | ≥22 |
|  | Tobramycin | 28 | S | ≥22 |
|  | Amikacin | 26 | S | ≥22 |
| Macrolide | Erythromycin | 19 | I | 18-20 |
| Tetracycline | Minocycline | 27 | S | 23 |
| Polymixin B | Colistin | 12 | S | ≥15 |
| Trimethoprim-Sulfamethoxazole | Co-Trimoxazole | 22 | S | ≥17 |
| Aminocoumarin | Novobiocin | 12 | R | ≥16 |


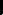


**Table S1**. **Antibiotic sensitivity profiling of *S. saprophyticus* against different antibiotic classes.**

Symbol S - Sensitive, R – Resistance, I – Intermediate. The zone of inhibition was measured and interpreted according to CLSI and EUCAST guidelines.

**Antibiotic sensitivity and resistance profile of all the strains used in tropism study**


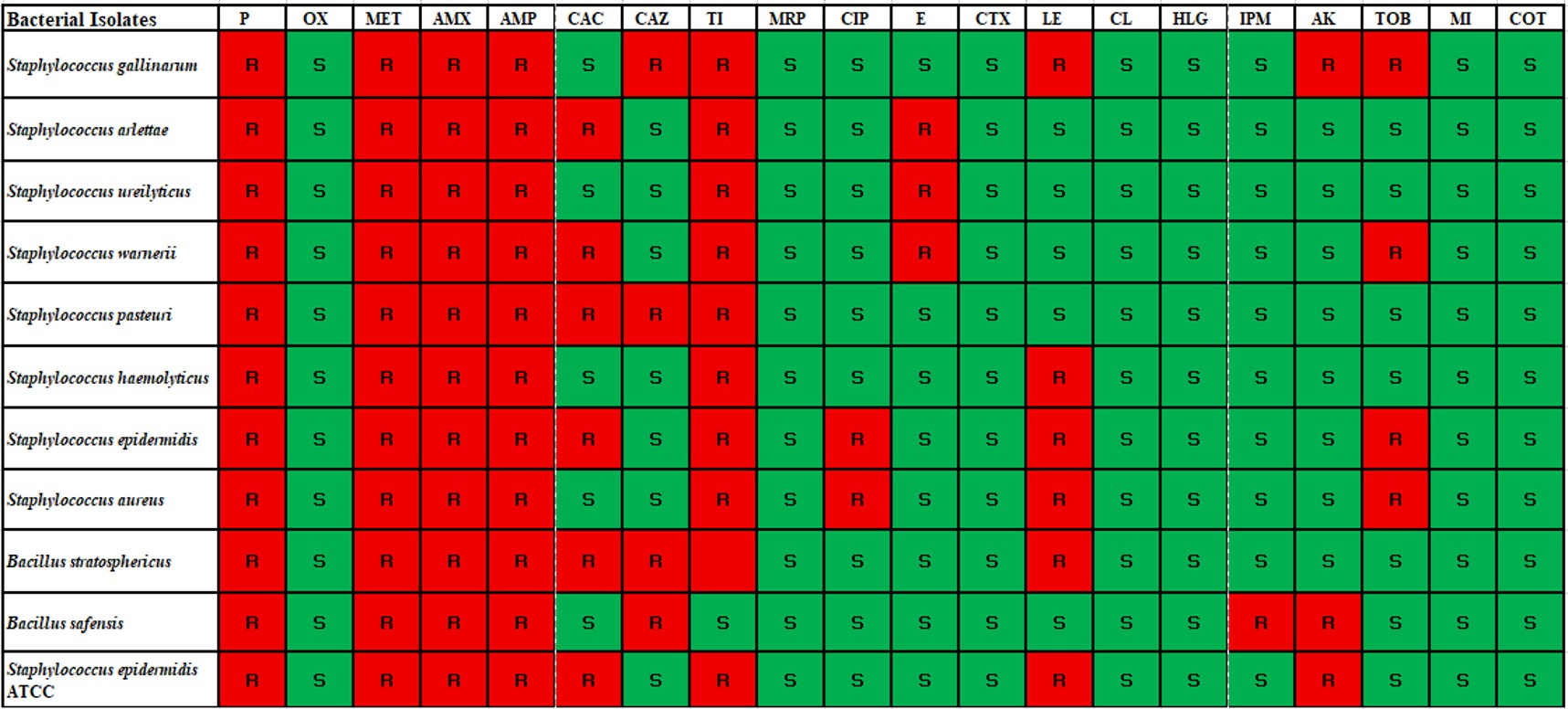


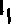


**Table S2**. **Antibiotic sensitivity profiling of *Staphylococcus strains used for tropism study* against different antibiotic classes.**

Symbol S – Sensitive (green colour), R – Resistance (red colour), I – Intermediate (yellow). The zone of inhibition was measured and interpreted according to CLSI and EUCAST guidelines

**Mitomycin C (Prophage) induction assay**


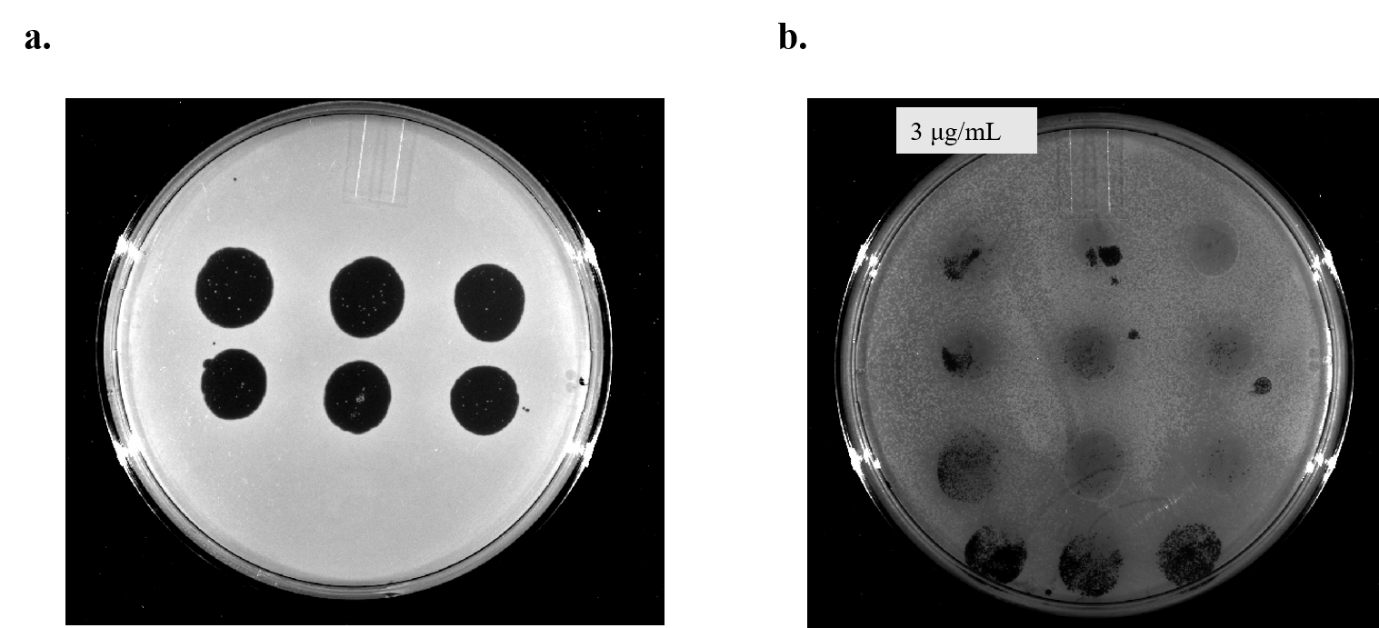


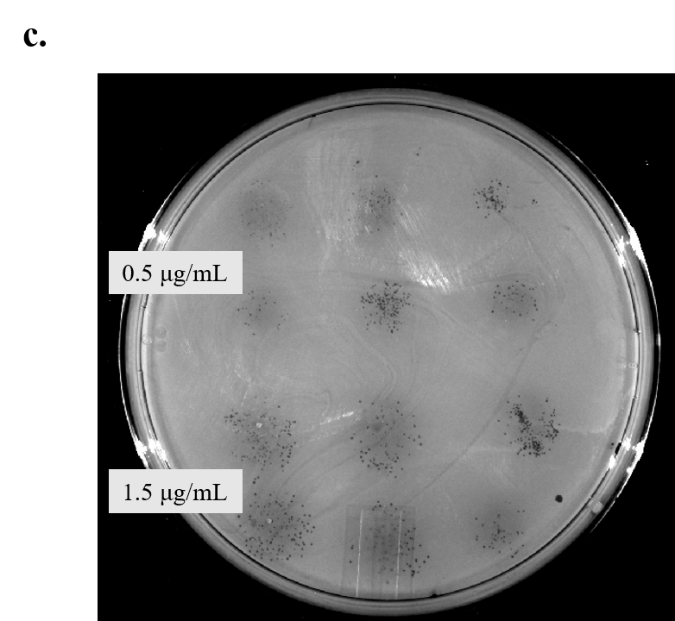


**Figure S2**. Spot assay of *S. saprophyticus* treated with different mitomycin C concentrations indicated zone of hazy clearance confirming the presence of prophages. (a) ØPh_SS01 spotted as positive control. (b) Host bacteria treated with 3 µg/mL mitomycin C. (c) 0.5 and 1.5 µg/mL mitomycin concentrations on *S. saprophyticus*.

**Phage-mediated reduction of *S. saprophyticus* in MS broth (liquid medium assay)**
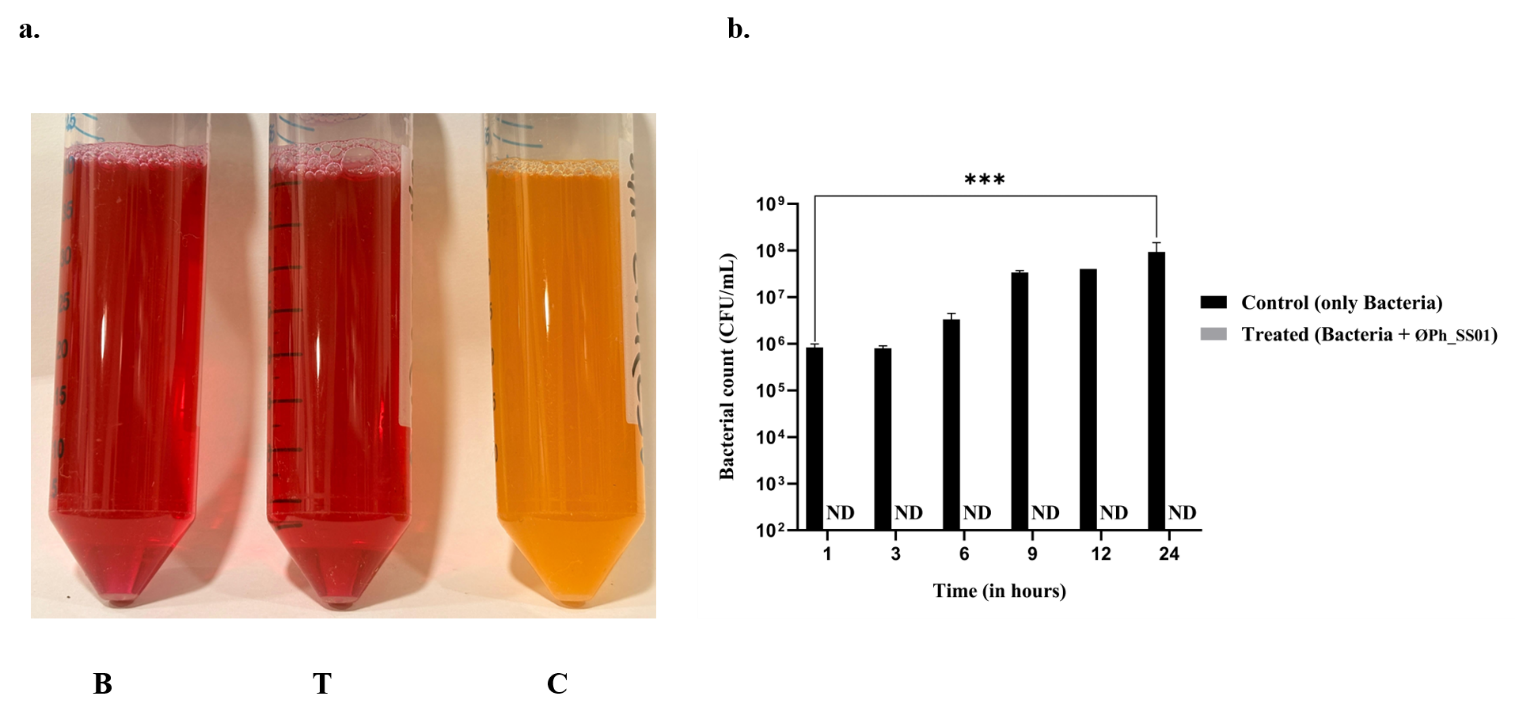


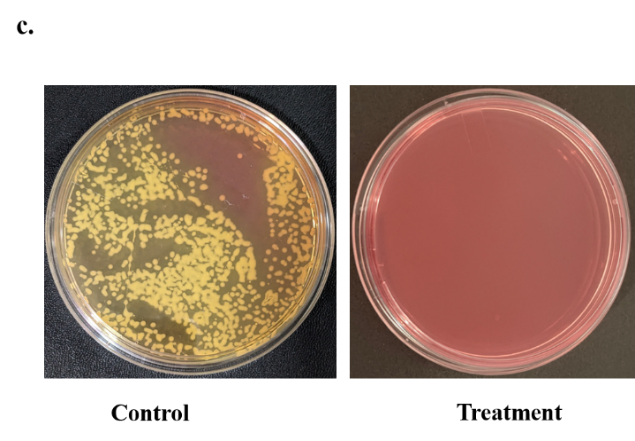


**Figure S3.** Phage-mediated targeting of *S. saprophyticus* in MS broth. (a,b) The blank (B), treated (T) and control (C) groups presenting phage-mediated bacterial reduction. In the control (yellow) group, increased bacterial growth and metabolism were observed compared to the treated (pink) group, which showed limited growth and retained a pink colour as that of the blank group. (c) The CFU/mL count of *S. saprophyticus* in control and treated. The bacterial count in CFU/mL was determined in control and treated over a period of 24 h. ND, not detected.
